# Efferocytosis in Health and Disease

**DOI:** 10.1002/mco2.70546

**Published:** 2025-12-14

**Authors:** Chaofu Li, Yukun Yang, Fating Zhou, Qiuyan Jiang, Yingying Jiang, Xuanjie Huang, Yiqiong Zhang, Zhengmeng Ye, Gang Xu, Guoying Kao, Ke Zhou, Fan Yang, Jun Xiao, Wei Wu, Chuanwei Li

**Affiliations:** ^1^ Department of Cardiology Chongqing Key Laboratory of Emergency Medicine Chongqing University Central Hospital (Chongqing Emergency Medical Center) College of Bioengineering Chongqing University Chongqing China; ^2^ Central Laboratory of Chongqing Emergency Medical Center Chongqing University Central Hospital School of Medicine Chongqing University Chongqing China; ^3^ Department of Neurology University Hospital Essen University of Duisburg‐Essen Essen Germany; ^4^ Intensive Care Unit Chongqing University Central Hospital (Chongqing Emergency Medical Center) College of Bioengineering Chongqing University Chongqing China; ^5^ Stomatology Major in School of Stomatology Xinjiang Medical University Urumqi China

**Keywords:** apoptotic cell clearance, biomarker‐ and artificial intelligence‐based monitoring, efferocytosis, immunometabolic rewiring, precision efferocytosis‐targeted therapy, TAM receptors

## Abstract

Efferocytosis is the fundamental mechanism by which phagocytes clear apoptotic cells to maintain tissue homeostasis. This process is also closely linked to immune tolerance, metabolic reprogramming, inflammation resolution, and tissue repair. In recent years, research spanning cardiovascular disease, autoimmune disorders, metabolic inflammation, neurodegeneration, and cancer has revealed diverse context‐dependent regulatory networks, including “eat‐me” and “don't‐eat‐me” signals, phagocytic receptors, intracellular signaling pathways, and metabolic checkpoints. Disruption of these regulatory layers contributes to the defective resolution of inflammation, persistent immune activation, and impaired tissue regeneration. However, a unified comparative framework that integrates these mechanisms across different disease states is lacking. In this review, we provide a comprehensive overview of the biology of efferocytosis, from apoptotic cell recognition and engulfment to downstream immunometabolic rewiring. We highlight disease‐specific alterations in atherosclerosis, myocardial infarction, autoimmune diseases, neuroinflammation, and the tumor microenvironment. In addition, we summarize the emerging therapeutic strategies, including receptor agonists, metabolic interventions, engineered extracellular vesicles, and immune checkpoint modulation. Finally, we propose a “full‐cycle” monitoring strategy that integrates imaging‐based quantification, circulating biomarkers, multiomics profiling, and artificial intelligence to enable dynamic assessment of efferocytosis in vivo.

## Graphical abstract

Efferocytosis in health and disease. Efferocytosis integrates apoptotic cell recognition, engulfment, digestion, and reprogramming to link cell clearance with tissue repair. Molecular checkpoints balance “eat‐me” signals such as phosphatidylserine against inhibitory “don't‐eat‐me” cues like CD47, while TAM receptors, scavenger receptors, and integrins coordinate cytoskeletal remodeling for phagocytic uptake. Digestion involves phagosome–lysosome fusion and LC3‐associated phagocytosis, which couple cargo degradation to macrophage metabolic rewiring via PPARγ–LXR and mTOR–AMPK pathways. These events drive anti‐inflammatory and reparative phenotypes, ensuring tissue homeostasis across cardiovascular, neural, pulmonary, metabolic, autoimmune, and oncological settings. Translational strategies include biomarker‐based monitoring and therapeutic modulation through CD47–SIRPα blockade or TAM receptor activation, underscoring efferocytosis as both a biological safeguard and a therapeutic target. *Abbreviations*: PS, phosphatidylserine; TAM, Tyro3/Axl/MerTK receptors; PI3K, phosphatidylinositol‐3‐kinase; LAP, LC3‐associated phagocytosis; PPARγ, peroxisome proliferator‐activated receptor‐γ; LXR, liver X receptor; AI, artificial intelligence; SIRPα, signal regulatory protein‐α.

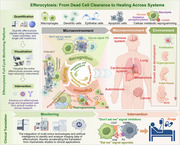

## 1 Introduction

**FIGURE 1:**
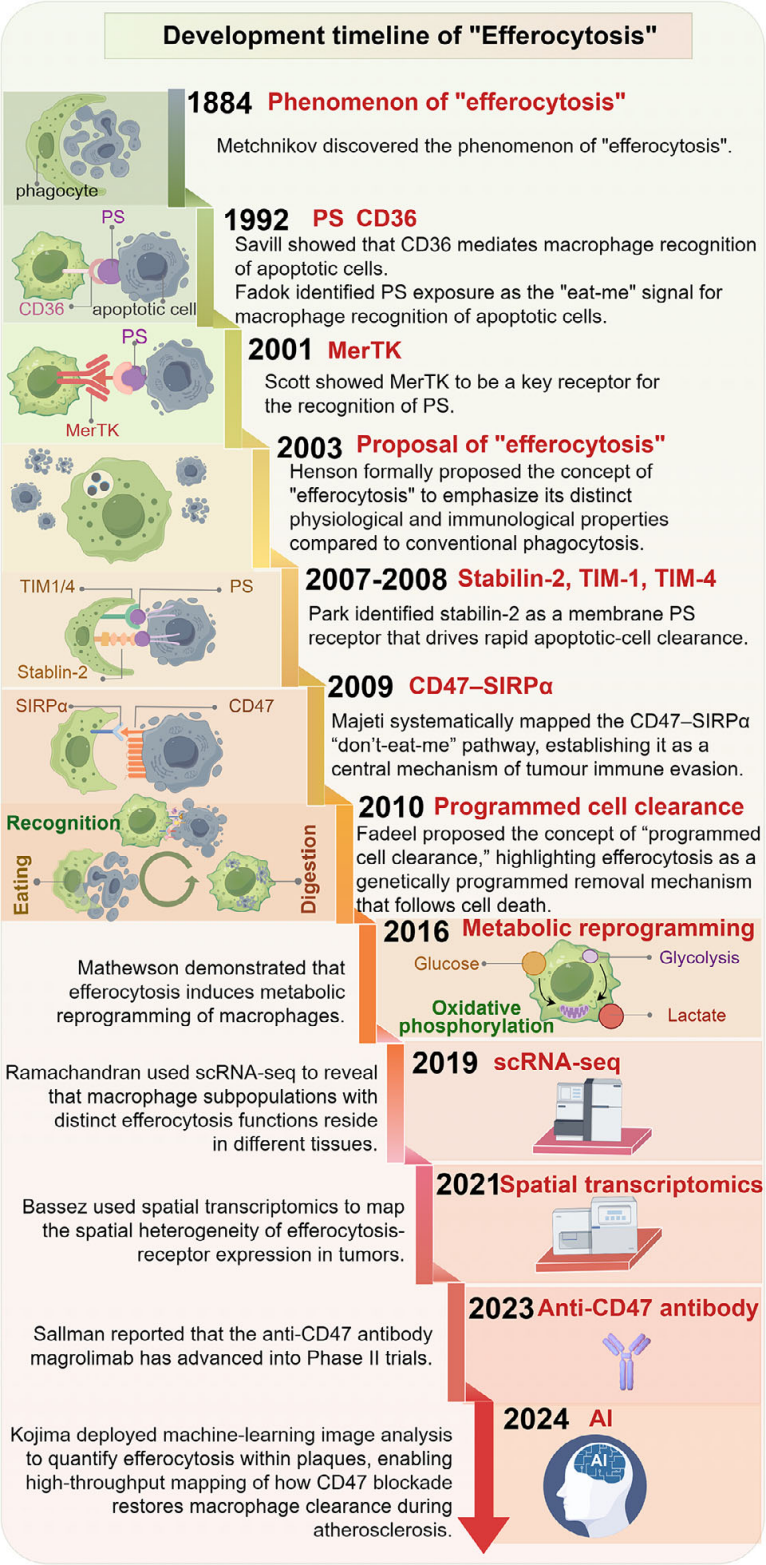
Development timeline of efferocytosis. Key milestones in efferocytosis research are summarized chronologically. The concept originated from Metchnikov's observations in 1884, followed by the identification of phosphatidylserine exposure and CD36‐mediated clearance in the early 1990s. MerTK was established as a phosphatidylserine receptor in 2001, and the term “efferocytosis” was formally proposed in 2003. Subsequent discoveries included Stabilin‐2, TIM‐1, and TIM‐4 as additional receptors, the CD47–SIRPα checkpoint pathway (2009), and the concept of “programmed cell clearance” (2010). Advances since 2016 have emphasized macrophage metabolic reprogramming, while technologies such as single‐cell RNA sequencing and spatial transcriptomics have refined understanding of efferocytic heterogeneity. Clinical translation is highlighted by anti‐CD47 antibodies entering Phase II trials (2022), and artificial intelligence applications (2024) provide opportunities for predictive modeling and precision therapy. *Abbreviations*: PS, phosphatidylserine; CD36, cluster of differentiation 36; MerTK, MER proto‐oncogene tyrosine kinase; TIM, T‐cell immunoglobulin and mucin domain; SIRPα, signal regulatory protein‐α; scRNA‐seq, single‐cell RNA sequencing; AI, artificial intelligence.

Efferocytosis is a specialized form of phagocytosis that refers to the selective removal of apoptotic cells by professional phagocytes, such as macrophages and dendritic cells, as well as certain “nonprofessional” phagocytes, including epithelial cells. In contrast to classical phagocytosis, efferocytosis typically prevents the triggering of inflammation, promotes immune tolerance, and preserves tissue homeostasis [1, 2]. The term “efferocytosis” was introduced by Henson in 2003 to highlight its distinct physiological and immunological features compared with conventional phagocytosis [3], although the underlying concept can be traced back to Metchnikov's seminal observations [4] (Figure 1). Over the past two decades, efferocytosis has been increasingly recognized not merely as a terminal clearance step but as a critical regulatory switch that determines whether inflammation resolves or persists and whether tissues repair or progress toward fibrosis [5]. This shift highlights the importance of elucidating the molecular signals and pathways that orchestrate efferocytosis.

At the mechanistic level, apoptotic cells display “eat‐me” signals—most prominently exposed phosphatidylserine (PS)—whereas viable cells present “don't‐eat‐me” signals such as cluster of differentiation 47 (CD47) [6, 7]. Together, these cues act as molecular checkpoints that regulate recognition and enforce the immune balance [1, 8, 9]. “Programmed cell clearance” further positions efferocytosis as a late programmed stage downstream of cell death [10, 11]. Central to this process are the Tyro3, Axl, and Mer receptor tyrosine kinases (TAM) and their bridging ligands, growth arrest‐specific 6 (GAS6) and Protein S, which orchestrate target recognition and engulfment [12, 13]. These receptors cooperate with scavenger receptors (SRs) and integrins to drive phagocytic cup formation, cytoskeletal remodeling, and efferosome maturation [14]. In parallel, signaling pathways such as phosphatidylinositol 3 kinase–protein kinase B (PI3K–AKT) and mammalian target of rapamycin–AMP‐activated protein kinase (mTOR–AMPK) regulate metabolic reprogramming, resolution of inflammation, and immune re‐education [15, 16, 17]. These cascades intersect with emerging processes including lipid mediator biosynthesis, macrophage polarization, and autophagy‐related phagocytosis, reinforcing the view that efferocytosis is a central node that links cell death, immune regulation, and tissue regeneration [18, 19]. Collectively, these mechanisms provide a biological basis for physiological protection and the pathological consequences outlined below.

Under physiological conditions, efferocytosis prevents the progression of apoptotic cells to lysis and secondary necrosis, thereby limiting uncontrolled inflammation and supporting tissue repair [19]. When efferocytosis is impaired, apoptotic cells and cellular debris accumulate and initiate or amplify pathological responses [20, 21]. In the cardiovascular system, defective efferocytosis promotes necrotic core expansion within atherosclerotic plaques and sustains chronic inflammation, thereby increasing the risk of plaque rupture and adverse events [22]. It also aggravates myocardial remodeling and compromises contractile function, contributing to myocardial infarction (MI) [23]. In the nervous system, inefficient clearance of dying neurons intensifies tissue injury and accelerates functional decline [24]. In an autoimmune context, impaired efferocytosis facilitates the release of self‐antigens and disrupts immune tolerance [8]. Within the tumor microenvironment, it establishes an immunosuppressive niche that fosters immune evasion and tumor progression [25, 26]. Collectively, these systemic consequences highlight efferocytosis as indispensable for homeostasis and emphasize its role as a central regulatory node in the pathogenesis, progression, and therapeutic targeting of diverse diseases.

From a translational perspective, interventions targeting the “don't‐eat‐me/eat‐me” axis and its downstream signaling pathways are advancing from mechanistic studies toward clinical application. Therapeutic strategies such as CD47–signal regulatory protein alpha (SIRPα) blockade and Mer tyrosine kinase/Axl receptor tyrosine kinase (MerTK/Axl) agonists have already entered early‐phase clinical trials [27], showing promising benefits in cancer immunotherapy, atherosclerosis (AS), and tissue repair. Nevertheless, broader clinical translation remains limited by several barriers, including a lack of standardized functional assays, insufficient pharmacokinetic (PK)/pharmacodynamic (PD) and biomarker frameworks, and persistent gaps between animal models and human diseases. To address these challenges, this review will (i) systematically delineate stage‐specific molecular networks and signaling pathways of efferocytosis; (ii) integrate insights from metabolomics, spatial transcriptomics, and artificial intelligence (AI) to unravel pathological circuits and therapeutic targets across cardiovascular, neurological, oncological, and autoimmune diseases; and (iii) propose a “full‐cycle efferocytosis” framework for dynamic monitoring and quantitative assessment. Such a framework is expected to facilitate patient stratification, harmonize clinical endpoints, and inform adaptive trial design, thereby providing a coherent roadmap that bridges basic mechanisms with clinical translation and lays the foundation for precision therapies.

## 2 Molecular Basis and Regulatory Pathways of Efferocytosis

**FIGURE 2:**
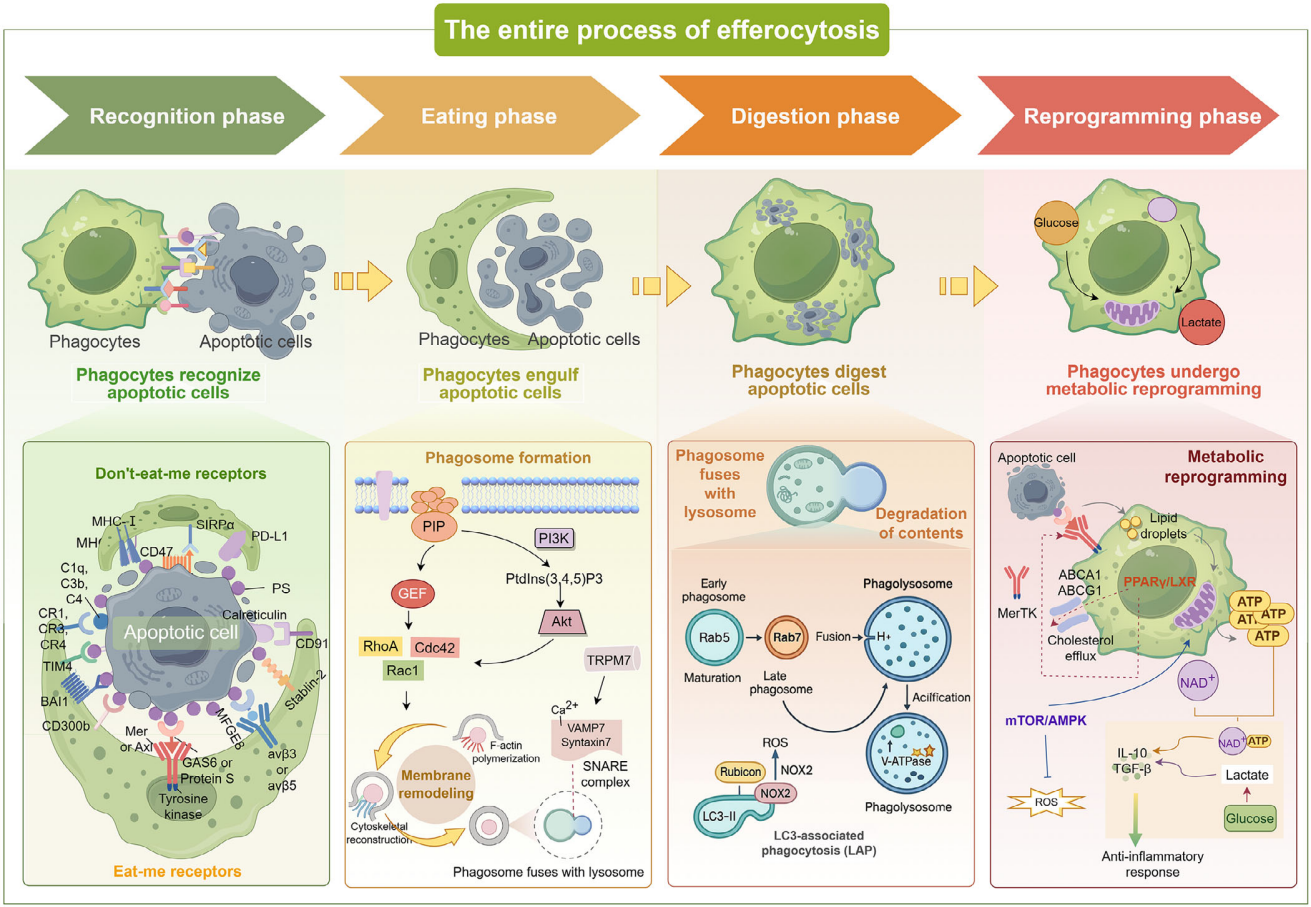
The entire process of efferocytosis. Efferocytosis proceeds through four interconnected phases: recognition, engulfment, digestion, and reprogramming. During recognition, apoptotic cells display “eat‐me” signals (e.g., phosphatidylserine, calreticulin) opposed by inhibitory “don't‐eat‐me” cues (CD47–SIRPα, PD‐L1). Engulfment requires TAM receptors, scavenger receptors, and integrins, with PI3K–AKT signaling, Rho GTPases, and calcium influx driving cytoskeletal remodeling and phagosome formation. Digestion involves phagosome–lysosome fusion, acidification, and enzymatic degradation, coordinated by LC3‐associated phagocytosis, Rubicon, and NOX2. In the reprogramming phase, metabolites from apoptotic cargo reshape phagocyte metabolism, promoting a shift from glycolysis to oxidative phosphorylation and enhancing lipid handling through PPARγ–LXR and mTOR–AMPK pathways. This leads to anti‐inflammatory cytokine release (IL‐10, TGF‐β) and reparative polarization. Efficient coordination ensures rapid, noninflammatory clearance, homeostasis, and inflammation resolution, while disruption results in defective clearance, secondary necrosis, and disease progression. *Abbreviations*: PS, phosphatidylserine; TAM, Tyro3/Axl/MerTK receptors; PI3K, phosphatidylinositol‐3‐kinase; AKT, protein kinase B; LAP, LC3‐associated phagocytosis; PPARγ, peroxisome proliferator‐activated receptor‐γ; LXR, liver X receptor; mTOR, mammalian target of rapamycin; AMPK, AMP‐activated protein kinase; NOX2, NADPH oxidase 2; IL‐10, interleukin‐10; TGF‐β, transforming growth factor‐β.

Efferocytosis is essential for maintaining tissue homeostasis, limiting inflammation, and promoting immune tolerance [28]. Under physiological conditions, large numbers of apoptotic cells are generated daily. If not promptly cleared, they can undergo secondary necrosis, leading to inflammation and immune dysregulation [29]. Efferocytosis occurs in three stages: recognition (find), engulfment (eat), and digestion (digest) [30] (Figure 2). During recognition, “eat‐me” and “don't‐eat‐me” signals dynamically regulate whether clearance is initiated. Engulfment depends on cytoskeletal remodeling and the formation of phagocytic cups and phagosomes, which enable apoptotic cells to be encapsulated and internalized [31]. Digestion involves phagosome–lysosome fusion and may trigger metabolic reprogramming [32]. Although these stages unfold sequentially, each stage is governed by distinct molecular pathways and is strongly shaped by the local microenvironment. Integration of efferocytic signals not only dictates the efficiency of apoptotic cell clearance but also influences the characteristics of the inflammatory response and the outcome of tissue repair [33]. Therefore, the following sections will examine the molecular pathways that operate at each stage and their dynamic regulation in health and disease. Against this background, we next examine recognition‐layer cues, beginning with “eat‐me” signals.

### 2.1 Recognition Phase

#### 2.1.1 “Eat‐me” Signals

During the recognition phase, apoptotic cells expose “eat‐me” signals that actively instruct phagocytes to initiate clearance [34, 35]. Among these signals, PS is the most classical and best characterized [36, 37]. Loss of membrane lipid asymmetry during apoptosis causes PS to translocate to the outer leaflet of the plasma membrane, where it acts as a critical marker for recognition and removal by phagocytes such as macrophages [14, 38]. PS can be recognized through both direct and indirect mechanisms. Direct recognition occurs via receptors including CD300b, BAI1, TIM4, and Stabilin‐2. Indirect recognition involves bridging proteins such as GAS6 and Protein S, which link PS to TAM family receptors (MerTK, Axl, and Tyro3) and activate downstream phagocytic signaling [39, 40]. In addition, accessory ligands, such as complement component 1q, complement component 3b, complement component 4, and surfactant protein a/d, enhance PS recognition efficiency and act cooperatively to promote the effective clearance of apoptotic cells [39, 41]. Taken together, these mechanisms establish PS as a central tag for identifying apoptotic cells.

In addition to PS, calreticulin (CRT) represents another crucial “eat‐me” signal. When exposed to the surface of dying cells, CRT binds to CD91 on phagocytic membranes, thereby initiating engulfment [42]. Beyond these classical ligands, recent studies have identified a variety of additional “eat‐me” signals. For example, extracellular vesicles (EVs) derived from M1 macrophages display the surface peptide RS17, which reprograms tumor‐associated macrophages (TAMs) into an immune‐active phenotype and enhances their capacity to eliminate tumor cells [43]. Similarly, EVs secreted by colorectal cancer cells are enriched in milk fat globule‐EGF factor 8 (MFGE8), which activates αvβ3 integrin on macrophages and improves their efficiency in clearing apoptotic cancer cells [44]. Extending this concept, Du et al. [45] engineered exosomes by incorporating a liver sinusoidal endothelial cell (LSEC)‐targeting peptide into lipid membranes, enabling selective uptake by LSECs and broadening strategies for delivering “eat‐me” signals. Collectively, these findings highlight EVs as versatile carriers and modulators of “eat‐me” signaling in both physiological and pathological contexts.

PS also shows considerable potential for nanoscopic imaging applications. Radiolabeled molecular probes that combine PS‐binding peptides with Zn (II) complexes display favorable PK properties, including faster blood clearance and superior imaging contrast compared with annexin V [46]. These advantages provide a new strategy for the PET‐ and SPECT‐based molecular imaging of apoptosis. Collectively, these probe designs illustrate the translational potential of PS targeting for in vivo detection of apoptosis.

In addition to imaging applications, advances in receptor biology have broadened PS recognition on the receptor side. Among these, cluster of differentiation 300f (CD300f) and Stabilin‐2 represent notable additions. Interestingly, CD300f exhibits cell‐type‐specific roles; it promotes efferocytosis in macrophages but suppresses this process in dendritic cells [1, 47].

The regulation of “eat‐me” signals is highly complex, extending beyond their basic role in apoptotic cell clearance [48]. “Eat‐me” signals are not only essential for the efficient clearance of apoptotic cells but also play crucial roles in macrophage polarization, tissue repair, and the maintenance of immune homeostasis [49]. When multiple “eat‐me” signals are presented simultaneously, phagocytes display remarkable selectivity in their recognition processes [50]. This selectivity is determined not only by receptor–ligand affinity, but also by factors such as signal strength, receptor expression patterns, cell type, and the surrounding microenvironment [51]. Importantly, these signals can interact in either synergistic or antagonistic ways, further shaping recognition outcomes. In this context, receptors with higher binding affinities are preferentially engaged in phagocytosis, whereas those with lower affinities are competitively excluded [52, 53]. This hierarchical engagement defines the priority of signal utilization and ultimately orchestrates the biological outcomes of efferocytic responses. These principles provide a conceptual bridge to subsequent discussions of “don't‐eat‐me” signals and their crosstalk with “eat‐me” pathways.

#### 2.1.2 “Don't‐eat‐me” Signals

During the recognition phase of efferocytosis, viable cells—unlike apoptotic cells—express “don't‐eat‐me” signals that safeguard immune homeostasis and prevent inappropriate phagocytosis [54]. Among these inhibitory pathways, the CD47–SIRPα axis is the best characterized [55]. CD47, broadly expressed on healthy cell membranes, engages SIRPα on macrophages to activate SHP1/SHP2‐mediated phosphatase signaling [56]. This cascade suppresses cytoskeletal rearrangements and inhibits phagocytic activation. Through this interaction, macrophages can distinguish apoptotic from viable cells, thereby maintaining tissue integrity.

In addition to CD47, other inhibitory pathways participate in the regulation of efferocytosis. Programmed death‐ligand 1 (PD‐L1), for example, attenuates T‐cell activation by binding to programmed death‐1 (PD‐1) on lymphocytes and directly impairs macrophage phagocytosis through PD‐1 engagement [57]. Moreover, classical major histocompatibility complex class I (MHC‐I) molecules downregulate natural killer cell cytotoxicity, whereas nonclassical MHC‐I variants contribute to immune evasion by modulating CD8⁺ T‐cell surveillance [58]. Collectively, these mechanisms act in concert with the CD47–SIRPα axis, broadening the spectrum of “don't‐eat‐me” signals across both innate and adaptive immune compartments.

Mounting evidence has indicated that malignant cells use these inhibitory pathways to evade immune‐mediated clearance. In particular, aberrant overexpression of CD47 is a hallmark of immune escape in multiple tumor types, effectively suppressing macrophage‐mediated efferocytosis [59, 60, 61]. To counteract this therapeutic strategy, monoclonal antibodies targeting CD47, such as magrolimab, have advanced into clinical trials for diverse hematological and solid malignancies [62]. These agents restore the efferocytic capacity and limit tumor progression. Although their efficacy as monotherapies is modest, combinatorial regimens incorporating chemotherapeutic agents, immune checkpoint inhibitors, and nanoparticle‐based delivery systems have demonstrated synergistic effects [63, 64]. For instance, magrolimab combined with the hypomethylating agent azacitidine has encouraging safety and preliminary efficacy data in patients with TP53‐mutated acute myeloid leukemia (AML) [65].

In addition to monoclonal antibodies, next‐generation immunotherapeutic strategies have also been actively explored. Zhang et al. [66] developed dual‐targeting nanoparticles that simultaneously inhibited CD47 and PD‐L1, thereby enhancing macrophage and cytotoxic T cell activation and inducing durable antitumor immune memory after radiotherapy. Additionally, novel inhibitory axes were identified. For example, the CD24–Siglec‐10 interaction has emerged as a pivotal mechanism that suppresses innate immune responses and facilitates immune evasion in various solid tumors [65]. These findings highlight the mechanistic complexity and redundancy of antiphagocytic signaling and support the rationale for multitargeted combination‐based immunotherapies.

Despite clinical progress in targeting “don't‐eat‐me” signals, critical gaps remain in understanding their context‐dependent integration and regulation. Within the tumor microenvironment, these inhibitory pathways seldom act in isolation, but instead form a dynamic and interconnected network that coordinates immune escape [67, 68]. A major challenge is selectively neutralizing these signals in malignant cells while preserving immunological tolerance in normal tissues. Future studies should aim to define the in vivo hierarchy, spatiotemporal dynamics, and crosstalk among distinct inhibitory pathways. Such efforts will advance our understanding of efferocytic regulation and guide the rational design of more selective, safe, and durable antitumor immunotherapies.

#### 2.1.3 Mechanical Force Recognition

As receptor‐mediated pathways of phagocytosis have become increasingly well‐defined, research attention has shifted toward the role of mechanical forces in regulating efferocytosis. Mechanobiology provides critical insights into how physical cues influence both the efficiency and specificity of apoptotic cell clearance and helps explain the mechanisms underlying dysfunction in disease [69]. During apoptosis, progressive cytoskeletal disassembly increases cell stiffness (quantified by the Young's modulus), thereby altering the mechanical signals perceived by phagocytes. These changes act in concert with classical “eat‐me” signals and serve as additional determinants of target recognition. Supporting this concept, experimental studies have shown that macrophages preferentially recognize and engulf stiffer apoptotic cells [70], highlighting the contribution of their biophysical properties to phagocytic selectivity.

### 2.2 Engulfment Phase

Following the recognition of “eat‐me” signals, phagocytes rapidly initiate cytoskeletal remodeling and phagosome formation to internalize dying cells. This step represents the central execution phase of efferocytosis that bridges upstream signal recognition with downstream degradation and metabolic reprogramming. The effectiveness and precision of this process are vital to preserve tissue homeostasis and regulating inflammation [71].

Engulfment begins with the direct contact between phagocytes and the target cell membrane. This interaction activates receptor‐mediated signaling cascades that drive actin cytoskeleton rearrangement and extension of pseudopodia, ultimately enclosing apoptotic cells within the phagosome [72]. The process is orchestrated by a complex network of regulatory mechanisms, including phagocytic receptors, small GTPases, calcium signaling pathways, and mechanotransduction systems, all functioning in a highly coordinated manner [73, 74]. To understand how efferocytosis is executed and how it can be impaired under pathological conditions, this section comprehensively examines the regulatory mechanisms governing the engulfment phase. Specifically, we focused on three interconnected levels: receptor engagement, intracellular signaling pathways, and biomechanical processes.

#### 2.2.1 Classification and Regulation of Engulfment Receptors

The engulfment phase is initiated by the precise recognition and binding of apoptotic cells through various receptors expressed on the surface of phagocytes. These receptors serve a dual function: they detect “eat‐me” signals—such as PS—and simultaneously activate downstream signaling pathways that drive cytoskeletal remodeling and phagosome formation, thus establishing the molecular framework of efferocytosis [75].

Based on their ligand specificity and functional roles, engulfment receptors are broadly categorized into three major classes: TAM receptor family, SRs, and integrins. The TAM receptor family, comprising Tyro3, Axl, and MerTK, acts as the central regulatory axis in efferocytosis. These receptors recognize PS on the surface of apoptotic cells through their ligands, GAS6 and Protein S, thereby facilitating phagocytic uptake [76, 77]. MerTK is predominantly expressed in the resting macrophages and functions as a tolerogenic receptor that supports the clearance of apoptotic cells under immunosuppressive conditions. In contrast, Axl is upregulated in response to inflammatory stimuli and plays a prominent role in mediating efferocytosis in inflamed tissues [78].

SRs are crucial mediators of lipid sensing and efferocytosis regulation [79]. For example, SR‐A1 directly interacts with Tyro3 and synergistically enhances the phagocytic capacity [80]. SR‐B1 promotes the recognition and clearance of oxidized low‐density lipoprotein (ox‐LDL) by activating the Src/PI3K/Rac1 signaling cascade, thereby contributing to the stabilization of atherosclerotic plaques and limiting the expansion of necrotic cores [81]. CD36, a prototypical lipid‐binding receptor of the SR family, binds to PS on apoptotic cells, modulates inflammatory signaling, and facilitates M2 macrophage polarization. Collectively, these functions enable CD36 to shape the disease microenvironment via multiple coordinated mechanisms [82, 83].

Integrins constitute another essential class of engulfment receptors, particularly the subtypes αvβ3, αvβ5, and αMβ2 (also known as CR3) [84, 85]. These integrins mediate adhesion between phagocytes and apoptotic cells by binding to bridging molecules such as MFGE8 or extracellular matrix (ECM) components, thereby promoting close membrane contact [86]. This interaction subsequently activates intracellular signaling cascades that drive cytoskeletal remodeling and facilitate phagosome formation [87]. Thus, integrin activation is tightly coupled with actin reorganization, which is indispensable for the successful internalization of apoptotic cells and the completion of efferocytosis.

#### 2.2.2 Signal Transduction and Cytoskeletal Remodeling

The activation of phagocytic receptors triggers rapid and coordinated cytoskeletal remodeling, enabling membrane protrusion and invagination to form pseudopodia and internalize the target cells into phagosomes. This process is orchestrated by small GTPases, phosphoinositide signaling, and downstream effectors [88]. One of the earliest steps involves the localized accumulation of phosphatidylinositol phosphates (PIPs) on the inner leaflet of the plasma membrane, which recruits guanine nucleotide exchange factors (GEFs) [89]. These GEFs activate small GTPases, such as Rac1, Cdc42, and RhoA. Rac1 plays a pivotal role in actin polymerization, driving pseudopod extension, and phagocytic cup formation [90]. Cdc42 primarily regulates cell polarity and directional movement, and works in concert with Rac1 to coordinate cytoskeletal rearrangement [91]. RhoA is transiently activated during the initial stage of cup formation but is subsequently downregulated to prevent excessive contractility, which could hinder efficient engulfment [92].

Closely intertwined with these events, the PI3K/AKT signaling axis serves as a central regulator of efferocytosis. Upon activation, PI3K catalyzes the conversion of PIP2 to PIP3 [PtdIns(3, 4, 5)P3], leading to AKT activation [93]. Activated Akt subsequently triggers small GTPase signaling cascades that further drive actin cytoskeletal remodeling, reinforcing the formation of phagocytic cups and target cell engulfment [94, 95]. Supporting this role, the transient receptor potential melastatin 7 (TRPM7) channel is essential for Ca^2^⁺‐dependent signaling; its loss disrupts phagosomal acidification and impairs the subsequent degradation of apoptotic cargo [96].

Despite the central role of these molecular signaling pathways in efferocytosis, the actual occurrence and efficiency of engulfment are ultimately determined by the dynamic interplay between “eat‐me” and “don't‐eat‐me” signals. This regulatory mechanism ensures target specificity and maintains immune tolerance under normal physiological conditions.

#### 2.2.3 Advances in Mechanobiological Regulation

Recent studies have shown that macrophages, as the primary effectors of phagocytosis, sense the mechanical properties of their microenvironments. Among mechanosensitive components, the ion channel Piezo1 plays a particularly prominent role. Piezo1 detects changes in extracellular stiffness and triggers Ca^2^⁺ influx, which activates downstream signaling pathways that promote cytoskeletal remodeling and pseudopod extension. In models of senescent red blood cell clearance, Piezo1‐mediated mechanotransduction enhances macrophage recognition and the engulfment of stiffer targets [97]. Notably, integrin‐generated forces are indispensable during the macrophage engulfment of target cells, supplying the traction necessary to initiate and propagate the phagocytic cup. When phagocytosis is frustrated, integrin‐mediated tension is rerouted to the cup, erecting a dynamic mechanical barricade that excludes CD45, a phosphatase that dampens engulfment signals, and thus ensures successful cup maturation and enlargement [98]. By complementing this extrinsic mechanosensing, the intrinsic cytoskeletal programs within macrophages are crucial for efficient efferocytosis. For example, splenic marginal zone macrophages require the transcriptional regulator, megakaryoblastic leukemia 1 (MKL1), to sustain F‐actin polymerization and remodeling. The loss of MKL1 reduces cytoskeletal tension, impairs pseudopod formation, and compromises apoptotic cell clearance [99].

Extending these mechanistic insights, the “mechanical matching” hypothesis proposes that substantial biophysical mismatches—such as differences in deformability or stiffness—between phagocytes and their targets can destabilize receptor–ligand interactions, thereby blunting downstream signal amplification and impeding phagocytic cup formation [53]. This mismatch may help explain the persistence of apoptotic cells in diseases such as cancer and AS, despite the presence of “eat‐me” cues. More broadly, mechanobiological inputs, phagocytic receptor signaling, and cytoskeletal control functions are integrated networks that ensure the efficiency and specificity of efferocytosis. Understanding the dynamic interplay between mechanosensing and signal transduction may reveal novel therapeutic targets for fine‐tuning efferocytosis, alleviating chronic inflammation, and facilitating tissue repair.

### 2.3 Digestion and Postengulfment Reprogramming

After recognizing and engulfing apoptotic cells, macrophages enter the digestion and reprogramming phase, a pivotal stage in efferocytosis in which cellular degradation is closely coupled with functional transformation. During this phase, apoptotic cells are sequestered within phagosomes, which fuse with lysosomes to initiate enzymatic degradation. Metabolites released through this process reshape the macrophage metabolism and phenotype, thereby promoting resolution and tissue repair [100, 101].

This phase is regulated by two major mechanisms. First, the fusion of phagosomes with lysosomes and the activation of lysosomal hydrolases ensure the efficient breakdown of cellular components [102]. Second, postengulfment metabolic reprogramming and signal transduction guide macrophages toward a reparative phenotype by modulating pathways such as glycolysis, lipid metabolism, and cholesterol efflux. Several key signaling cascades—including LC3‐associated phagocytosis (LAP), the peroxisome proliferator‐activated receptor gamma–liver X receptor (PPARγ–LXR) axis, and the mTOR–AMPK pathway—coordinate these changes [103, 104]. Disruption of these pathways may impair apoptotic cell clearance, resulting in persistent inflammation, immune imbalance, or disease development.

Therefore, the digestion phase not only facilitates cellular clearance but also acts as a metabolic switch that links efferocytosis to macrophage reprogramming. Elucidating the regulatory mechanisms governing this phase is essential for understanding how immune homeostasis is maintained, and may uncover promising therapeutic targets for the treatment of inflammation‐related diseases.

#### 2.3.1 Phagosome–Lysosome Fusion and Cargo Degradation

As a fundamental step in the digestion phase, macrophages must efficiently degrade engulfed apoptotic cells to ensure effective efferocytosis [105]. This degradation begins with the fusion of phagosomes and lysosomes. Phagosome maturation is initiated by a small GTPase Rab5, which regulates early phagosome formation and vesicular trafficking. As maturation progresses, Rab7 replaces Rab5, promoting the transition to late‐stage phagosomes, and establishing the molecular and structural prerequisites for lysosomal fusion [106]. This fusion process depends on the coordinated action of SNARE protein complexes and membrane‐anchored proteins such as lysosome‐associated membrane protein 1, which mediates the precise docking and merging of phagosomal and lysosomal membranes, ultimately forming degradative phagolysosomes.

Following fusion, V‐ATPase is activated in the phagolysosomal membrane, where it drives proton translocation and acidifies the intraluminal environment. The acidic environment activates a range of lysosomal hydrolases, including cathepsins, which efficiently degrade key apoptotic cell components (proteins, lipids, and nucleic acids) [107, 108]. Timely degradation of these substrates prevents prolonged antigen exposure and limits local inflammation [106].

Notably, efferocytic degradation is not a simple replica of canonical autophagy but is governed by distinct yet interconnected regulatory pathways. One such pathway is LAP, which plays a pivotal role in efferocytosis. Unlike conventional autophagy, in which LC3‐II is recruited to double‐membrane autophagosomes, LAP is defined as the conjugation of LC3‐II to single‐membrane phagosomes [109].

This process is regulated by Rubicon, which exerts a dual control. Rubicon activates NADPH oxidase 2 (NOX2), promoting the generation of reactive oxygen species (ROS) that modify the phagosomal membrane and facilitate LC3 recruitment. In contrast, Rubicon inhibits autophagosome maturation, maintaining phagosomes in a state favorable for efficient degradation [110]. Local ROS production not only enhances phagosome–lysosome fusion but also augments the degradative capacity of phagosomes [111]. In contrast, Rubicon or NOX2 deficiency disrupts LAP, leading to defective apoptotic cell clearance, accumulation of cellular debris, and development of chronic inflammation or autoimmune pathologies [110].

In summary, efficient efferocytic degradation relies on three integrated processes: phagosome–lysosome fusion, acidification of the intraluminal space, and regulation by LAP. Together, these events establish a structural and functional basis for the metabolic reprogramming of macrophages and the maintenance of immune homeostasis.

#### 2.3.2 Postengulfment Metabolic Reprogramming

Following the engulfment of apoptotic cells, macrophages not only remove cellular debris, but also undergo a dynamic phase of metabolic reprogramming that promotes inflammation resolution and the restoration of tissue homeostasis [112, 113, 114]. This reprogramming is marked by a temporal shift in energy metabolism. Glycolysis is transiently upregulated in the early phase to rapidly generate ATP for energy‐intensive processes such as phagosome formation and endosome fusion. As the degradation of apoptotic components proceeds, macrophages transition toward mitochondrial oxidative phosphorylation (OXPHOS), thereby improving energy efficiency and dampening the prolonged activation of proinflammatory pathways [100]. In parallel, metabolic reprogramming influences macrophage polarization, with proinflammatory subsets relying primarily on glycolysis and reparative subsets depending on OXPHOS [115]. In addition to macrophages, other cell types display distinct metabolic responses. For example, endothelial and epithelial cells upregulate the glucose transporter SLC2A1 after engulfing apoptotic cells, thereby increasing glucose uptake and aerobic glycolysis to ensure a sufficient ATP supply for sustained phagocytic activity [116, 117].

Parallel to this shift in energy metabolism, lipid handling by efferocytic macrophages undergoes significant changes [118]. During efferocytosis, macrophages internalize large quantities of lipids from apoptotic cells. These lipids are stored in lipid droplets and subsequently broken down via lipolysis and β‐oxidation to produce energy. To prevent cholesterol accumulation, which can induce endoplasmic reticulum stress and inflammatory responses, macrophages upregulate cholesterol transporters, such as ATP‐binding cassette sub‐family member 1 (ABCA1) and ATP‐binding cassette sub‐family g member 1 [100]. This enhances the cholesterol efflux, preserves membrane integrity, and supports cellular homeostasis. These lipid metabolic adaptations not only sustain the metabolic balance of phagocytes but also provide a foundation for their functional reprogramming.

In addition to energy production and lipid regulation, metabolic reprogramming also shapes the immunological phenotype of macrophages. Postefferocytic macrophages secrete anti‐inflammatory cytokines, including interleukin‐10 (IL‐10) and transforming growth factor‐beta (TGF‐β), whose expression depends on restored mitochondrial function and adequate NAD⁺ levels [119]. Concurrently, glycolysis‐derived lactate acts as a paracrine signal that promotes the polarization of neighboring macrophages toward anti‐inflammatory or proresolution phenotypes. This contributed to the establishment of a coordinated immunosuppressive microenvironment [100]. Thus, metabolic remodeling not only facilitates the intrinsic functional transition of efferocytic macrophages but also enables intercellular immunometabolic communication.

At the molecular level, these metabolic changes are orchestrated by several key signaling pathways. Among them, the PPARγ–LXR axis serves as a central regulator of lipid metabolism and cholesterol efflux [120]. Activation of PPARγ and LXR by apoptotic cells induces the expression of cholesterol transporters such as ABCA1 [121]. Additionally, LXR upregulates the expression of the MerTK receptor, enhancing macrophage recognition and the clearance of apoptotic cells. This forms a positive feedback loop— “efferocytosis–metabolism–enhanced clearance”—that promotes the resolution of inflammation [122]. The pharmacological activation of LXR enhances the efferocytic capacity and alleviates chronic inflammation [123].

In addition, the mTOR–AMPK signaling axis plays a crucial role as a metabolic sensor for modulating efferocytosis‐associated metabolic responses. Hyperactivation of mTOR suppresses efferocytosis and triggers lysosome‐dependent cell death, thereby disrupting cellular homeostasis [124]. In contrast, AMPK is activated in response to energy depletion or oxidative stress. It promotes fatty acid β‐oxidation, facilitates the clearance of ROS, and restores metabolic balance by reversing glycolytic dysregulation and inflammasome activation observed under defective efferocytosis. Collectively, these effects prevent secondary infections and limit inflammation exacerbation [125].

In summary, postengulfment metabolic reprogramming serves as a critical bridge between efferocytosis and restoration of tissue homeostasis.

## 3 The Role of Efferocytosis in Physiological Homeostasis

**FIGURE 3:**
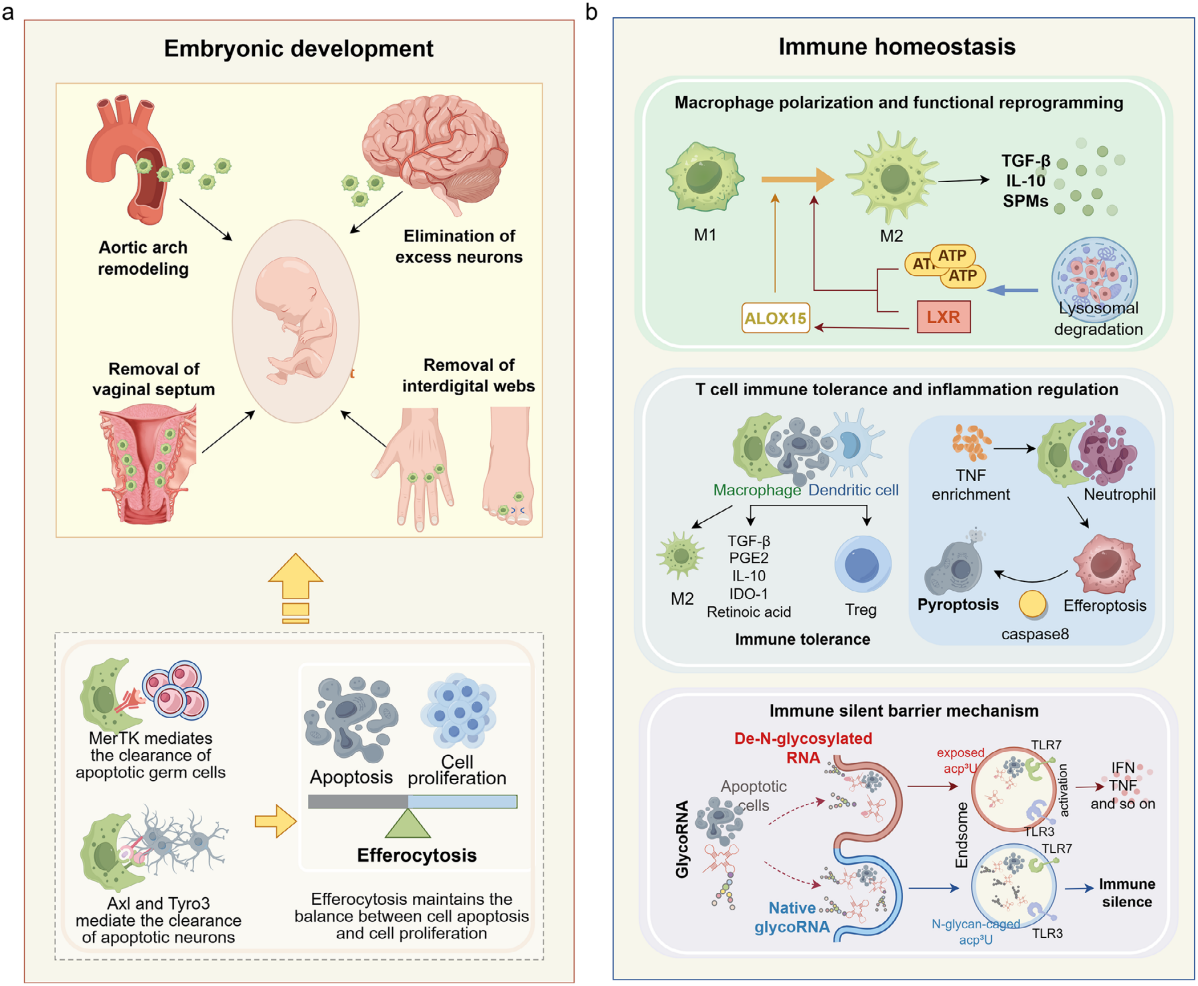
Physiological roles of efferocytosis in embryonic development and immune homeostasis. Efferocytosis is essential for morphogenesis and immune regulation. (a) In embryonic development, clearance of apoptotic cells enables noninflammatory remodeling, including aortic arch shaping, neuronal pruning, vaginal septum resorption, and interdigital web regression, with TAM receptors (MerTK, Axl, Tyro3) mediating germ cell and neuron removal. (b) In immune homeostasis, efferocytosis reprograms macrophages toward anti‐inflammatory states via the LXR–ALOX15 axis, promoting TGF‐β, IL‐10, and specialized proresolving mediators. It also induces Treg cells, restrains proinflammatory responses, and maintains an “immune silent barrier” through glycoRNA–TLR3/TLR7 signaling, thereby preventing autoimmunity and sustaining immune balance. *Abbreviations*: TAM, Tyro3/Axl/MerTK receptors; MerTK, MER proto‐oncogene tyrosine kinase; LXR, liver X receptor; ALOX15, arachidonate 15‐lipoxygenase; TGF‐β, transforming growth factor‐β; IL‐10, interleukin‐10; SPMs, specialized proresolving mediators; TLR, Toll‐like receptor; Treg, regulatory T cell.

Efferocytosis plays an essential role in embryonic development, organ formation, the establishment of immune tolerance, and the preservation of steady‐state conditions [126, 127] (Figure 3). Under normal conditions, many cells undergo programmed apoptosis. If not efficiently cleared, these apoptotic cells can trigger inflammation, disrupt the tissue architecture, and elicit aberrant immune responses. Efferocytosis ensures the rapid and noninflammatory removal of apoptotic cells, thereby preventing cellular “spillover” and maintaining the integrity of the local microenvironment. A systematic evaluation of efferocytosis across these physiological contexts not only deepens our understanding of its multifaceted roles in maintaining homeostasis but also offers a conceptual and mechanistic basis for exploring how its dysfunction contributes to disease pathogenesis.

### 3.1 Efferocytosis in Embryonic Development and Organogenesis

Programmed cell death is a fundamental mechanism in embryonic development and organogenesis and plays a pivotal role in shaping tissue architecture and regulating cell numbers. Developmental processes, such as aortic arch remodeling, elimination of excess neurons, vaginal septum resorption, and regression of interdigital webs, are orchestrated through spatially and temporally regulated apoptotic events [128]. However, apoptosis alone does not ensure tissue integrity, and prompt and efficient clearance of apoptotic cells is equally vital [129]. Efferocytosis facilitates this removal in a noninflammatory manner, thereby preventing the release of intracellular contents, suppressing inflammation, and maintaining the stability of the developmental microenvironment [130]. Therefore, the coordinated interplay between apoptosis and efferocytosis forms a crucial foundation for normal tissue morphogenesis (Figure 3a).

At the molecular level, the TAM receptor family, comprising Tyro3, Axl, and MerTK, is highly expressed in embryonic stem cells and plays an indispensable role in embryonic development [131]. Among these receptors, MerTK is critical for Sertoli cell‐mediated efferocytosis in the testes [132]. Sertoli cells, which line the seminiferous tubules, are responsible for clearing apoptotic germ cells and maintaining an environment conducive to differentiation. In MerTK‐deficient mice, this clearance process is impaired, leading to a disrupted testicular architecture and pronounced reproductive dysfunction, underscoring the vital role of efferocytosis in preserving organ structure and function [1]. Furthermore, Tyro3 is abundantly expressed in embryonic tissues. Its simultaneous deletion with Axl significantly increases the apoptosis of gonadotropin‐releasing hormone neurons during early development, resulting in central nervous system abnormalities. In adulthood, this manifests as delayed sexual maturation and irregular estrous cycles, which are indicative of reproductive neuroendocrine dysregulation [133].

The importance of efferocytosis extends beyond morphological development to immune regulation. During early development, the immune system develops tolerance to self‐antigens to prevent future autoimmune responses. In this process, macrophages clear apoptotic cells and release anti‐inflammatory cytokines such as TGF‐β and IL‐10. These cytokines suppress local inflammation and promote the differentiation of regulatory T (Treg) cells, thereby facilitating the establishment of peripheral immune tolerance [134]. The thymus, the primary site for central immune tolerance, relies on efferocytosis to eliminate self‐reactive T‐cell remnants during development. This clearance prevents them from escaping to the periphery and mitigates the risk of autoreactivity at an early stage [135].

Disruption of efferocytic function, such as through the deletion or dysfunction of TAM receptors, leads to the accumulation of apoptotic cells, which in turn triggers immune recognition and attack against self‐antigens. In mouse models, simultaneous deficiency of all three TAM receptors results in the production of autoantibodies, persistent inflammation, and progressive tissue damage, ultimately resembling a systemic lupus erythematosus‐like phenotype [136]. These findings underscore the essential role of an intact efferocytic system, beginning during embryonic development, in establishing immune tolerance and safeguarding against autoimmune disorders.

Efferocytosis plays a crucial regulatory role in embryonic and neonatal development. It is essential not only for proper organ morphogenesis but also for establishing and maintaining immune homeostasis. These physiological roles provide a fundamental framework for understanding the mechanisms by which efferocytic dysfunction leads to disease development.

### 3.2 The Role of Efferocytosis in the Regulation of Immune Homeostasis

Efferocytosis is critical for preserving tissue integrity and for coordinating physiological functions by efficiently removing apoptotic cells during embryonic development and tissue maintenance. However, in mature organisms, the immune system must adapt to the increasingly complex and dynamic internal and external stimuli. As a result, efferocytosis evolved beyond a simple clearance mechanism to serve as a central regulator of immune equilibrium (Figure 3b). Efferocytosis is essential for maintaining immune tolerance and preventing the development of chronic inflammatory and autoimmune disorders. It acts not only as the final step in apoptotic cell disposal but also as a trigger for macrophage reprogramming and T cell fate decisions. This section explores the major pathways through which efferocytosis regulates immune homeostasis, focusing on three key aspects: macrophage polarization, induction of T‐cell tolerance, and formation of an “immunologically silent” microenvironment. Together, these mechanisms underscore the multilayered and hierarchical nature of efferocytic regulation in immune control and offer a conceptual foundation for understanding how efferocytic dysregulation contributes to immune‐mediated diseases.

#### 3.2.1 Macrophage Polarization and Functional Reprogramming

Macrophages are essential for maintaining immune homeostasis, particularly because of their capacity for functional reprogramming after engulfing apoptotic cells. This process promotes their polarization toward an anti‐inflammatory M2‐like phenotype, which is marked by elevated production of IL‐10, TGF‐β, and specialized proresolving mediators (SPMs) such as resolvins, lipoxins, and maresins. These anti‐inflammatory and proresolving factors work together to facilitate tissue repair and actively resolve inflammation [18, 19].

In addition to serving as phagocytic scavengers, postefferocytic macrophages have emerged as central immune regulators through coordinated metabolic and functional reprogramming. Specifically, lysosomal degradation of apoptotic cell components enables the recycling of bioactive macromolecules, including carbohydrates, amino acids, lipids, and nucleotides, to meet the anabolic and energetic demands of these cells. This metabolic adaptation not only supports macrophage survival, but also reinforces their polarization toward an anti‐inflammatory and tissue‐reparative phenotype [128].

LXR pathway is a key signalling axis underlying this process and plays a pivotal regulatory role during efferocytosis. LXR enhances anti‐inflammatory responses by modulating gene transcription and upregulating downstream targets such as lipoxygenase arachidonate 15‐lipoxygenase (ALOX15), thereby promoting the synthesis of SPMs during the resolution phase of inflammation [137]. Furthermore, LXR signaling intersects with major metabolic pathways, including cholesterol metabolism and OXPHOS, further driving macrophages toward a proresolving and regenerative phenotype.

Taken together, these findings highlight that efferocytosis not only terminates apoptotic cell clearance but also initiates macrophage reprogramming and phenotypic transformation, thereby supporting immune homeostasis and laying the groundwork for the effective resolution of inflammation and tissue regeneration.

#### 3.2.2 T Cell Immune Tolerance and Regulation of Inflammation

Efferocytosis contributes to immune homeostasis by promoting anti‐inflammatory macrophage responses and modulating adaptive immunity through antigen presentation and immunosuppressive mediator release. Following the engulfment of apoptotic cells, macrophages and dendritic cells secrete immunoregulatory factors such as TGF‐β and prostaglandin E2 (PGE2), which suppress proinflammatory cytokine production and help establish a tolerogenic immune environment [18]. In addition, these phagocytes release IL‐10, retinoic acid, and IDO1, which collectively promote Treg cell differentiation of Treg cells [138]. Notably, at mucosal barrier sites such as the intestinal epithelium and lungs, areas with active efferocytosis exhibited elevated frequencies of Treg cells, highlighting the critical role of apoptotic cell clearance in the maintenance of local immune tolerance [139, 140].

Beyond fostering immune tolerance, efferocytosis also mitigates inflammation by clearing senescent neutrophils, thereby protecting tissues from secondary injury [141]. However, the consequences of efferocytosis are context‐dependent and can be shaped by the surrounding inflammatory microenvironment. For instance, under conditions of elevated tumor necrosis factor (TNF), efferocytosis may transition from a homeostatic to a proinflammatory state—a phenomenon termed “efferoptosis” [142]. In such settings, macrophages that engulf neutrophils may undergo caspase‐8‐dependent, but NLRP3 inflammasome‐independent pyroptosis.

Interestingly, the blockade of T cell immunoglobulin and mucin‐domain‐containing protein 3 can reduce splenic macrophage efferoptosis and improve survival in TNF‐induced systemic inflammatory response syndrome; however, it simultaneously exacerbates injury in parenchymal organs, such as the lungs and kidneys. These observations highlight the tissue‐specific effects of efferocytosis, which is typically protective in parenchymal tissues, but potentially detrimental to lymphoid organs. Further evidence suggests that the functional consequences of efferocytosis are modulated by the genetic background of the host. For example, Tnfr1‐deficient mice exhibited enhanced tolerance in a model of Escherichia coli‐induced septic peritonitis. In these animals, macrophages retain their efferocytic capacity while evading activation‐induced cell death, thereby reducing excessive systemic inflammation.

Taken together, these findings reinforce the idea that efferocytosis contributes to immune tolerance by modulating antigen presentation and promoting Treg cell induction of Treg cells. Moreover, it plays a dynamic and context‐dependent role in balancing the protective and proinflammatory immune responses. Its immunoregulatory effects are orchestrated by the interplay between tissue microenvironmental cues, intracellular signaling pathways, and host genetic variation.

#### 3.2.3 Mechanisms of the Immunologically Silent Barrier

Efferocytosis process promotes inflammation resolution through macrophage polarization toward the M2 phenotype and the induction of anti‐inflammatory mediators such as IL‐10 and TGF‐β. Crucially, this also depends on the molecular mechanisms that prevent inappropriate immune recognition of self‐derived components. Recent studies have uncovered a novel regulatory mechanism termed the “immunologically silent barrier, ” in which RNA N‐glycosylation plays a pivotal role in shielding self‐RNA during efferocytosis [143]. Normal cells generate a unique nucleoside modification, 3‐(3‐amino‐3‐carboxypropyl) uridine (acp3U)—synthesized by enzymes such as DTWD2. These modified nucleosides can be conjugated with sialylated N‐glycans to form glycoRNAs. Upon apoptotic cell engulfment, the glycan moiety of glycoRNA acts as a chemical shield that masks self‐RNA from endosomal pattern recognition receptors TLR3 and TLR7. This prevents the activation of downstream TBK1–IRF3 and NF‐κB signaling cascades, thereby inhibiting the production of type I interferons and proinflammatory cytokines. This ensures that apoptotic cells are cleared in an immunologically silent manner, thereby preserving their self‐tolerance.

Further evidence has revealed that the disruption of this glycan barrier, for instance, by PNGase F‐mediated deglycosylation, leads to the exposure of acp3U, which in turn activates endosomal TLRs and triggers robust innate immune responses. Interestingly, even the retention of a single N‐acetylglucosamine residue on glycoRNA is sufficient to maintain immune quiescence. These findings suggest that RNA glycosylation represents a previously underappreciated layer of immunoregulation that functions alongside canonical “eat‐me” signaling and postengulfment metabolic reprogramming. Taken together, these insights expand the conceptual framework of efferocytosis. They revealed that apoptotic cell clearance is governed by a multilayered system that integrates surface cues, intracellular signaling, and the epitranscriptomic landscape of nucleic acids.

## 4 Efferocytic Dysfunction in Disease Pathogenesis

Efferocytosis is fundamental for maintaining tissue integrity and immune balance. When this process is disrupted, the consequences extend across multiple organ systems, including the cardiovascular, nervous, and respiratory systems, leading to metabolic, autoimmune, and malignant diseases. Impaired clearance of apoptotic cells leads to the accumulation of cellular debris, excessive release of damage‐associated molecular patterns (DAMPs), sustained inflammation, and dysregulated immune responses, all of which aggravate tissue injury and disease progression. In the following sections, we examine how defective efferocytosis contributes to disease pathogenesis across these systems and summarize the therapeutic approaches targeting specific stages of the efferocytic process: recognition, engulfment, and digestion.

### 4.1 Efferocytic Dysfunction in Cardiovascular Diseases

Under pathological conditions, dysfunction in any phase of efferocytosis, recognition, engulfment, or digestion can result in the accumulation of cellular debris and release of DAMPs. This in turn triggers secondary necrosis and sustains chronic inflammation. The cardiovascular system is particularly vulnerable to such dysfunction owing to its high metabolic demands and constant exposure to hemodynamic stress.

**FIGURE 4:**
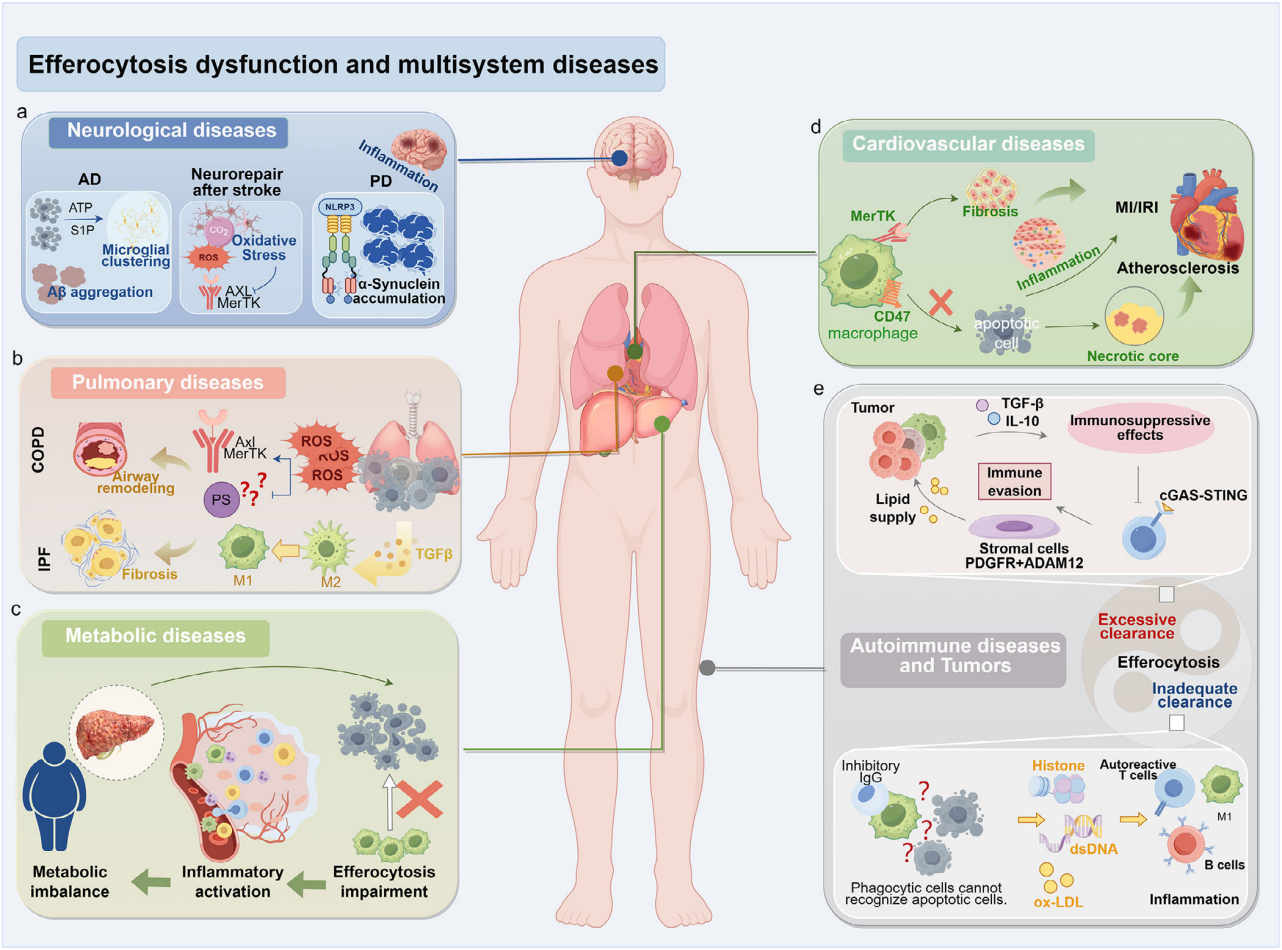
Efferocytosis dysfunction in multisystem diseases. Impaired or excessive efferocytosis contributes to the pathogenesis of diverse diseases by disrupting immune homeostasis and tissue repair. (a) Nervous system: defective clearance promotes β‐amyloid and α‐synuclein accumulation in AD and PD, and aggravates stroke injury. (b) Cardiovascular system: insufficient efferocytosis accelerates necrotic core expansion, fibrosis, and inflammation in atherosclerosis, MI, and IRI. (c) Pulmonary system: impaired clearance contributes to airway remodeling in COPD and fibrosis in IPF. (d) Metabolic disorders: defects amplify inflammation in obesity and fatty liver disease. (e) Immune and tumors: inadequate clearance exposes self‐antigens and breaks tolerance, while tumors exploit efferocytosis‐derived signals (TGF‐β, IL‐10, PDGF, ADAM12) for immune evasion and stromal remodeling. *Abbreviations*: AD, Alzheimer's disease; PD, Parkinson's disease; MI, myocardial infarction; IRI, ischemia–reperfusion injury; CHF, chronic heart failure; COPD, chronic obstructive pulmonary disease; IPF, idiopathic pulmonary fibrosis; TAM, Tyro3/Axl/MerTK receptors; TGF‐β, transforming growth factor‐β; IL‐10, interleukin‐10; PDGF, platelet‐derived growth factor; ADAM12, a disintegrin and metalloproteinase domain‐containing protein 12.

Increasing evidence has implicated defective efferocytosis in the progression of cardiovascular diseases, including AS and MI with reperfusion injury (Figure 4a). Inadequate clearance of dying cells impairs immune cell reprogramming and disrupts proreparative signaling pathways, thereby exacerbating inflammation and structural damage. Elucidation of these mechanisms may lead to the identification of novel biomarkers and therapeutic targets. This section explores the role of efferocytic dysfunction in cardiovascular disease pathogenesis and discusses emerging strategies aimed at restoring effective clearance mechanisms.

#### 4.1.1 Atherosclerosis

In AS, inefficient clearance of apoptotic cells results in their accumulation within vascular lesions, promoting the formation of necrotic cores. These necrotic cores exacerbate local inflammation and compromise the plaque stability, thereby increasing the risk of adverse cardiovascular events [144]. Efferocytosis dysfunction plays a central role in this pathological cascade and arises from multiple mechanisms, including the upregulation of “don't‐eat‐me” signals, metabolic dysfunction in macrophages, and the suppression of key phagocytic receptors such as MerTK by proinflammatory cytokines within the lesion microenvironment [145, 146, 147, 148]. And studies have shown that macrophage clearance of apoptotic cells (efferocytosis) can promote the activation of anti‐inflammatory signaling pathways to maintain plaque stability. This is because nucleotides derived from the hydrolysis of apoptotic cell DNA by phagolysosomal DNase2a can activate the DNA–PKcs–mTORC2/Rictor pathway, which increases Myc to promote noninflammatory macrophage proliferation [149].

Building on this mechanistic understanding, recent studies have explored therapeutic strategies aimed at correcting efferocytic dysfunction in AS. For example, anti‐CD47 antibodies block the “don't‐eat‐me” signal, thereby promoting apoptotic cell clearance, reducing early plaque formation and enhancing the stability of existing plaques in apoE‐deficient mouse models [145]. However, apoptotic foam cells and concentrated cholesterol render plaque macrophages with an overwhelming lipid burden, limiting the proefferocytosis effect of checkpoint blockade therapy in AS. Cai et al. [150] developed retinoic acid‐loaded macrophage membrane‐biomimetic liposomes (R@MLP) to enhance the efferocytotic ability of macrophages. Notably, the inherent presence of SIRPα on the R@MLP effectively blocks the interaction between CD47 on apoptotic cells and SIRPα on macrophages, thereby achieving CD47–SIRPα inhibition. In addition, Resolvin D1 (RvD1), a prototypical SPM, facilitates the removal of necrotic cells and improves the local immune microenvironment by reprogramming macrophage metabolism to enhance fatty acid oxidation and mitochondrial OXPHOS, underscoring its therapeutic potential [151]. Interventions that upregulate MerTK expression or prevent its proteolytic cleavage can restore the efferocytic capacity of macrophages and contribute to plaque stabilization [18, 118].

#### 4.1.2 MI and Ischemia–Reperfusion Injury

Like AS, MI and ischemia–reperfusion injury (MI/IRI) are characterized by extensive cardiomyocyte apoptosis and the release of DAMPs. These DAMPs activate and amplify local immune responses, causing secondary injury to adjacent healthy tissues and sustaining a vicious cycle of inflammation and tissue destruction [152]. When apoptotic cells are not efficiently cleared, they undergo secondary necrosis, releasing abundant proinflammatory mediators that worsen the inflammatory milieu and impede myocardial repair [153].

The principal reason for this defective clearance is dysregulated macrophage efferocytosis, which sustains inflammation and impairs tissue repair. Central to this dysfunction is impaired signaling through the TAM receptor family, particularly MerTK [154]. Following MI/IRI, MerTK expression is downregulated or cleaved by proteases such as ADAM17, preventing the transition of macrophages to a reparative CD11b⁺F4/80⁺Ly6C^lo^MerTK^hi^ phenotype and reducing their capacity to clear apoptotic cardiomyocytes. The resulting accumulation of necrotic debris and proinflammatory mediators exacerbates myocardial injury and promotes fibrosis progression [153]. In parallel, compromised TAM signaling disrupts postengulfment metabolic reprogramming, which normally induces fatty acid oxidation and mitochondrial electron transport, thereby prolonging inflammation and hindering regeneration [1]. Additionally, the expression of other efferocytosis‐related receptors, such as TREM2, is suppressed during MI/IRI, further diminishing efferocytic efficiency and regenerative capacity [155].

Against this backdrop, enhancing macrophage‐mediated clearance of apoptotic cardiomyocytes represents a promising strategy for breaking the pathological cycle [105]. Recent studies have proposed several strategies to enhance this process and improve MI/IRI outcomes. During apoptotic cell clearance, macrophages upregulate vascular endothelial growth factor C, which promotes lymphangiogenesis and facilitates cardiac repair, highlighting macrophage modulation as a therapeutic avenue [152]. Beyond its role in efferocytosis, TREM2 signaling supports cardiac regeneration through metabolic reprogramming. Notably, TREM2 agonists are in clinical trials for Alzheimer's disease (AD), providing a basis for potential cardiovascular applications [155]. Combination strategies also show added benefits; coactivation of MerTK with liposomal PEP‐20 (a CD47 antagonist) or exogenous macrophages with CCR2 (C‐C chemokine receptor type 2) enhances macrophage phagocytic capacity, reduces cardiac inflammation, and promotes tissue regeneration [156]. Similarly, combining SPMs with LXR agonists improved functional recovery in IRI models, underscoring a promising therapeutic direction [157]. Complementing these approaches, therapeutic delivery systems have emerged as tools to enhance efferocytosis in the heart. For example, using a lipid nanoparticle (LNP) delivery system to administer dual mRNA encoding lysosomal cysteine protease Legumain and antifibroblast activation protein (FAP) CAR directly to the infarct zone can reprogram macrophages on site, generating “efferocytosis‐enhanced, FAP‐targeted CAR‐MΦ, ” which significantly improves the treatment efficacy of MI fibrosis [158].

In conclusion, impaired efferocytosis is the central mechanism that sustains inflammation and limits tissue repair after MI/IRI. Therapeutic approaches that reprogram macrophage phenotypes and enhance apoptotic cell clearance offer promising opportunities to improve postinfarction recovery.

### 4.2 Efferocytic Dysfunction in Neurological Disorders

The mechanisms of impaired efferocytosis characterized by cardiovascular diseases, illuminate the link between chronic inflammation and tissue injury. Analogous mechanisms operate in neurological disorders, but often manifest with greater complexity and disease specificity (Figure 4b). Because the nervous system is extremely sensitive to perturbations in immune homeostasis, efferocytic imbalance may not only be a consequence of disease but also an initiating event that precipitates neuroinflammation and disrupts neural function. Studying efferocytic dysfunction under neurological conditions will deepen our understanding of its organ‐specific pathological significance.

#### 4.2.1 Alzheimer's Disease

Neuronal apoptosis is a major driver of pathology in AD and related neurodegenerative disorders. Under normal conditions, exposed “eat‐me” signals on apoptotic neurons are recognized by microglia, enabling rapid clearance. When efferocytosis is compromised, apoptotic neurons and their derivatives (apoptotic bodies) accumulate and create a persistent burden of cellular debris [159]. These remnants release chemotactic “find‐me” cues (e.g., ATP, S1P) that recruit additional microglia and amplify local inflammation [160]. Impaired clearance also promotes β‐amyloid (Aβ) deposition: Aβ plaques act as competitive sinks for “eat‐me” cues, diverting microglial recognition from apoptotic neurons [161], while the coexistence of Aβ deposits with apoptotic bodies provides a chronic inflammatory stimulus that skews microglia toward a proinflammatory state, increasing IL‐1β, TNF‐α, and other cytokines [160]. Together, Aβ and apoptotic debris synergistically activate the NLRP3 inflammasome, initiating downstream inflammatory cascades that further damage neuronal structure and function [161]. These events establish a mechanistic link between defective efferocytosis and increased neuroinflammation in patients with AD.

Phagocytic receptors including TREM2, MerTK, and CD36, are central to the efferocytic control of AD. Disease‐associated TREM2 variants (e.g., R47H) lower affinity for PS, thereby impairing microglial proliferation, migration, and Aβ plaque compaction and accelerating disease progression [162]. Conversely, the TREM2‐agonist monoclonal antibody AL002c increases microglial efferocytosis, enhances Aβ clearance, dampens neuroinflammation, and improves cognition in AD mouse models [163]. MerTK dysregulation likewise compromises efferocytosis, allowing apoptotic cells and Aβ deposits to accumulate, sustaining inflammation, and injuring neurons [164]. CD36, a receptor for oxidized PS, further modulates the removal of apoptotic cells and Aβ; its dysregulation heightens inflammatory signaling [165]. Ultimately, inadequate PS exposure or defective receptor recognition prevents efficient clearance, leading to the retention of apoptotic cells and the aggravation of neuroinflammation and cell death [162]. Collectively, these receptor‐level abnormalities highlight efferocytosis as a potential therapeutic target for AD.

Aducanumab, a monoclonal anti‐Aβ antibody, has advanced to Phase III clinical trials for AD and exerts its therapeutic effect by enhancing Aβ clearance [166]. In parallel, engineered microglia equipped with synthetic efferocytosis receptors are being developed to selectively phagocytose Aβ aggregates. These chimeric receptors fuse anti‐Aβ antibody fragments to efferocytic signaling domains, thereby markedly increasing Aβ clearance, reducing neurotoxicity, and improving cognitive performance in animal models [167]. Together, these translational approaches directly build on the mechanistic insights provided above, linking receptor biology with candidate therapies.

#### 4.2.2 Parkinson's Disease

Similar to AD, patients with Parkinson's disease (PD) exhibit marked defects in efferocytosis. The inefficient clearance of apoptotic dopaminergic neurons leads to the accumulation of cellular debris and DAMPs in the brain. This burden sustains the activation of microglia and astrocytes, drives chronic inflammation, exacerbates injury to the surviving neurons, and compromises neuronal regeneration and tissue repair [164].

In this inflammatory context, abnormal aggregation of α‐synuclein is a central driver of PD pathogenesis. These aggregates are directly neurotoxic and downregulate microglial receptors that recognize “eat‐me” signals, thereby impairing recognition and binding of apoptotic neurons and increasing the phagocytic burden [165]. In parallel, α‐synuclein aggregates activate the microglial NLRP3 inflammasome, eliciting robust secretion of proinflammatory mediators and further entrenching the inflammatory milieu [161]. Together, these effects suppress efferocytic clearance and skew microglia toward a proinflammatory state, compounding the loss of immunoregulatory and efferocytic functions. This feed‐forward loop provides a rationale for interventions aimed at restoring phagocytic clearance. To interrupt this loop, one approach employs annexin V, a PS‐binding protein that enhances the presentation of “eat‐me” signals to microglial receptors. By improving target recognition, annexin V restores microglial effector function and promotes phagocytic clearance of apoptotic neurons. In animal models of PD, annexin V increases efferocytic efficiency and ameliorates behavioral deficits, supporting its therapeutic potential [165]. Complementary to this strategy, activation of the AMPK/Gas6/Axl pathway alleviates macrophage senescence, restores efferocytic capacity, and attenuates both neuronal injury and astrocyte activation [168].

#### 4.2.3 Poststroke Neural Injury and Repair

Efferocytic defects contribute not only to chronic neurodegeneration but also to injury–repair dynamics following acute cerebrovascular events. During the acute phase of ischemic stroke, ischemia–hypoxia triggers widespread neuronal apoptosis and necrosis, releasing cellular debris and DAMPs [169]. The resulting microenvironment, marked by oxidative stress and elevated levels of inflammatory mediators, suppresses the expression and activity of phagocytic receptors (e.g., MerTK and AXL), thereby diminishing microglial recognition and clearance of apoptotic cells [170]. In this milieu, inflammatory cytokines such as IL‐1β and TNF‐α, together with DAMPs, further attenuate phagocytic signaling, creating a self‐reinforcing cycle of impaired efferocytosis and uncontrolled inflammation [159].

Owing to this impaired clearance, apoptotic cells persist and continue to release intracellular constituents and DAMPs. These danger cues sustain high levels of inflammatory cytokines (e.g., IL‐6, TNF‐α) and amplify local neuroinflammation [164]. The ensuing inflammatory milieu injures surviving neurons, compromises the blood–brain barrier integrity, enables peripheral immune cell infiltration, and worsens parenchymal damage. Efferocytic defects limit the production of anti‐inflammatory mediators and blunt reparative signaling, ultimately suppressing tissue repair and neurogenesis, thus perpetuating the cell cycle [160].

During the poststroke repair phase, restoration of efferocytosis is a pivotal driver of neuroregeneration. First, efficient debris clearance eliminates immunostimulatory cues in the infarct and peri‐infarct zones, promoting the polarization of microglia and infiltrating macrophages toward the M2/repair phenotype. These cells secrete IL‐10 and TGF‐β, establishing an immune milieu conducive to tissue reconstruction [159, 171]. In parallel, efferocytosis downregulates axonal growth inhibitors, fostering axonal regrowth and the recovery of functional neural circuits [160]. Downstream, activation of STAT6/Arg1 and PPARγ pathways further consolidates anti‐inflammatory and reparative programs, limits pathological ECM deposition, and facilitates neural remodeling [160].

Extending this paradigm beyond ischemic stroke, efferocytosis is also critical in subarachnoid hemorrhage (SAH) [164]. In this setting, extravasated erythrocytes within the subarachnoid space are the major drivers of post‐SAH brain injury, and their timely clearance dampens inflammation and neurotoxicity. Efferocytic pathways mediate the recognition and removal of these erythrocytes; when engaged, they help prevent secondary brain injury, underscoring the broad relevance of efferocytosis in stroke‐related neuroprotection [172].

Leveraging these insights, several strategies have been investigated to enhance efferocytosis after stroke. Nanoliposome delivery of RvD1 is a promising immunomodulatory approach: RvD1 crosses the blood–brain barrier, targets inflamed regions, and markedly increases apoptotic cell clearance in murine stroke models, thereby reducing tissue injury and improving neurological function [173]. In addition, overexpression of miR‐223 promotes poststroke brain repair, likely by regulating immune cell phenotypic switching and efferocytosis‐related signaling pathways, suggesting a therapeutic avenue with translational potential for ischemic stroke [174]. Collectively, these findings suggest that efferocytosis is a tractable target for poststroke neuroprotection and repair.

### 4.3 Efferocytic Dysregulation in Pulmonary Diseases

Unlike relatively protected organs, such as the heart and brain, the lungs are continuously exposed to airborne insults and a high‐oxygen milieu, placing exceptional demands on the speed and precision of apoptotic cell clearance. When efferocytosis is impaired, chronic inflammation and structural injury can develop and persist, initiating and subsequently amplifying pulmonary pathology. Accumulating evidence implicates efferocytic defects as key drivers of chronic obstructive pulmonary disease (COPD) and idiopathic pulmonary fibrosis (IPF), providing mechanistic insights and revealing new therapeutic opportunities for chronic lung diseases. This section first examines COPD and IPF, highlighting the common feedforward loops and emerging efferocytosis‐targeted interventions [175] (Figure 4c).

#### 4.3.1 Chronic Obstructive Pulmonary Disease

In COPD, the delayed clearance of apoptotic alveolar epithelial cells (AECs) leads to the accumulation of necrotic debris, which sustains inflammation and accelerates parenchymal destruction and airway remodeling [176]. Cigarette smoking and oxidative stress are the two drivers of impaired clearance. In smoking‐exposed animal models, the expression of key efferocytic receptors (MerTK and Axl) on alveolar macrophages is downregulated, which directly diminishes efferocytic efficiency, amplifies inflammation, aggravates tissue injury, and promotes disease progression [177]. In parallel, oxidative stress—another core pathogenic mechanism—further compromises efferocytosis: excessive ROS damage cellular membranes and inhibit PS externalization, limiting the display of “eat‐me” signals; ROS also impair mitochondrial function, reducing macrophage bioenergetic capacity and phagocytic activity, dysregulating signaling, and amplifying inflammatory cascades [101]. Together, these processes establish a feed‐forward loop, in which defective efferocytosis and chronic inflammation perpetuate progressive lung injury.

Against this pathophysiological backdrop, nanoparticle‐based approaches to restore macrophage efferocytosis are gaining momentum. For example, Chun et al. [178] engineered a biomimetic nanoparticle (ACM@U‐FGF21) that mimicked apoptotic cell membranes to enhance macrophage recognition and uptake, enabling targeted pulmonary delivery of fibroblast growth factor 21 (FGF21) and markedly reducing lung inflammation and injury. In parallel, nanodelivery platforms using SPMs or annexin A1 (Anxa1) also augment efferocytic capacity. In preclinical models, SPMs promote macrophage clearance of apoptotic cells and lower the inflammatory burden, whereas Anxa1 nanoparticles deliver the effector protein to macrophages, increase phagocytic activity, and restore alveolar homeostasis, indicating a precision medicine strategy for COPD [101]. These COPD‐focused advances illustrate the feasibility of efferocytosis‐centered interventions and set the stage for parallel strategies for fibrotic lung disease.

#### 4.3.2 Idiopathic Pulmonary Fibrosis

Complementing these COPD‐specific mechanisms in IPF, diverse stresses, such as microenvironmental disruption and recurrent injury, drive the apoptosis of AECs [179, 180]. Inefficient clearance of apoptotic AECs is an early trigger of fibrogenesis, which acts as a danger cue that activates local immune responses, aberrantly stimulates fibroblasts, and promotes excessive ECM deposition, thereby accelerating fibrosis [176, 181]. Aberrant activation of the TGF‐β signaling axis further aggravates this process by simultaneously suppressing efferocytosis and amplifying profibrotic signaling, directly promoting fibroblast transdifferentiation, skewing macrophage polarization, and downregulating phagocytic receptors, thereby destabilizing efferocytic feedback loops and establishing a self‐reinforcing cycle that links apoptosis, inflammation, and fibrosis [182].

Given this pathogenic framework and feed‐forward loop, accumulating evidence indicates that the therapeutic modulation of efferocytosis can slow and potentially reverse fibrogenesis. In animal models, engineered GAS6 proteins or Axl agonists, particularly when combined with antifibrotic agents, markedly enhance the macrophage‐mediated clearance of apoptotic cells, suppress fibroblast activation, and limit ECM deposition. Taken together, these findings suggest that efferocytosis is a tractable therapeutic target in IPF and supports the development of more precise immunomodulatory treatment strategies [182]. Collectively, these COPD data underscore efferocytosis as a unifying therapeutic axis for chronic lung diseases.

In summary, impaired efferocytosis drives the initiation and progression of chronic lung diseases such as COPD and IPF. When intact, efferocytosis modulates immune responses, preserves the alveolar microenvironment, and limits fibrotic remodeling; failure of efferocytosis disrupts these processes and worsens the disease course. Therefore, therapies that restore or augment efferocytosis, particularly when paired with early detection and precise targeting, represent a promising direction for future pulmonary treatments.

### 4.4 Efferocytic Dysregulation in Metabolic Diseases

Evidence from cardiovascular, neurological, and pulmonary disorders indicates that defective efferocytosis not only sustains chronic inflammation and tissue injury, but also destabilizes metabolic homeostasis, an underappreciated role (Figure 4d). Therefore, situating efferocytic dysfunction within the pathophysiology of metabolic diseases is both conceptually and translationally important [183].

Against this conceptual backdrop, sustained efferocytosis requires coordinated metabolic regulation and sufficient energy. Rapid activation of 6‐phosphofructo‐2‐kinase/fructose‐2, 6‐bisphosphatase 2 (PFKFB2) transiently augments macrophage glycolysis, and the ensuing lactate upregulates MerTK and LRP1, thereby supporting ongoing efferocytosis [184]. Conversely, extrinsic stressors and endogenous insults often perturb arginine metabolism, restrict tricarboxylic acid cycle flux, and impair mitochondrial function. These metabolic derangements destabilize macrophage bioenergetics, attenuate efferocytosis, sustain inflammation, and disrupt tissue homeostasis [185]. This metabolic framing provides a mechanistic bridge to disease contexts such as diabetes.

Reinforcing these observations, parallel evidence highlights the robustness of the efferocytosis‐related mechanisms. Defects in efferocytosis are particularly pronounced in diabetes and its associated complications. Diabetic wounds exhibit reduced M2 macrophages, accumulation of apoptotic neutrophils, and elevated methylglyoxal (MGO) [186]. MGO‐induced ROS activate p38 MAPK, upregulate the E3 ligase FBXO32, drive the ubiquitination and degradation of the transcription factor KLF4, and repress MerTK transcription by impairing phagocytosis and prolonging inflammation [186]. In addition, activated neutrophils release neutrophil extracellular traps (NETs) that suppress macrophage efferocytosis through the PI3K/Rac1 pathway, further delaying wound healing in diabetes [31]. This same mechanism contributes to chronic inflammation in diabetic periodontitis [187]. To date, several therapeutic modulators have been identified. Gasdermin E facilitates efferocytosis by reprogramming neutrophil death pathways, thereby promoting the resolution of inflammation [188]. Eldecalcitol restored macrophage phagocytic capacity and alleviated chronic periodontal inflammation [187]. At the epigenetic level, SIRT6 represses miR‐216/217, enhances the macrophage clearance of apoptotic neutrophils, reduces inflammation, and supports tissue repair [189].

Taken together, dysregulated efferocytosis sustains chronic inflammation in metabolic diseases, such as obesity and diabetes, by reshaping macrophage polarization, reducing phagocytic capacity, and disrupting tissue repair programs, serving as a pivotal link between metabolic imbalance and immune dysfunction. Accordingly, systematically charting efferocytosis‐coupled metabolic circuits, especially nodes governing the GAS6/MerTK axis, lactate metabolism, and mitochondrial homeostasis, may reveal therapeutic targets and enable precise interventions that attenuate inflammation, promote tissue repair, and improve outcomes in metabolic syndrome.

### 4.5 Dual Roles of Efferocytosis in Autoimmune Disease and Cancer

Efferocytosis is a tightly regulated clearance process that maintains immune homeostasis and inhibits inflammation. The consequences are highly context‐dependent and may even be opposing [190]. In autoimmune diseases, inefficient efferocytosis prolongs antigen availability and fuels persistent immune activation [191]. Within the tumor microenvironment, excessive or “silent” efferocytosis reduces tumor‐antigen exposure, blunts antitumor responses, and facilitates immune escape. This context‐specific dichotomy supports tailored interventions that enhance protolerogenic efferocytosis to re‐establish tolerance in autoimmunity, while limiting protumoral clearance to increase immunogenicity and strengthen antitumor immunity (Figure 4e).

#### 4.5.1 Efferocytic Impairment in Autoimmune Diseases

Under physiological conditions, efferocytosis rapidly removes apoptotic cells, thereby preventing antigen release, limiting the induction of inflammatory cytokines, and preserving immune tolerance. However, in autoimmune disorders such as Sjögren's syndrome, this process is impaired [192]. Reduced inhibitory IgG weakens the macrophage recognition of early apoptotic cells, leading to the retention of cellular debris in tissues and persistent activation of mucosal immunity [193]. The retained apoptotic cells undergo secondary necrosis and release dsDNA, oxidized lipids, and histones, which activate B cells through TLR7/9 and are cross‐presented by dendritic cells, promoting Th17 expansion and establishing a feed‐forward loop of autoantibody production and inflammation [138]. In parallel, insufficient apoptotic cues polarize macrophages toward an M1 phenotype, increasing TNF‐α and IL‐1β production and further amplifying tissue injury and inflammatory spread [194]. Taken together, defective clearance sustains autoantigen availability and proinflammatory polarization, providing a rationale for therapies that restore efferocytosis.

To counter this pathological process, researchers have developed biomimetic strategies to restore efferocytic homeostasis. Building on this rationale, Yuan et al. [195] engineered apoptotic cell‐mimicking nanovesicles that retain CD44‐ and Mac‐1‐mediated tissue homing and display the PS “eat‐me” signal. In rheumatoid arthritis and collagen‐induced arthritis models, these vesicles induced macrophage M2 polarization, reduced synovitis, and suppressed Th17‐driven inflammation. In a complementary approach, liquid‐nitrogen‐treated macrophages were repurposed as “cell‐corpse” drug carriers; because they are efficiently engulfed by autologous macrophages, they enabled targeted drug delivery and sustained long‐term remission in rheumatoid arthritis models, supporting a strategy for tolerance reconstitution [196].

#### 4.5.2 Modulation of Efferocytosis in the Tumor Immune Microenvironment

Extending this context‐dependence on cancer, tumors frequently co‐opt for efferocytosis within the tumor's immune microenvironment to enable immune escape [197]. Although dying cancer cells expose PS and other “eat‐me” cues that would normally promote immune activation, TAMs clear them rapidly [198]. This curtails tumor‐antigen availability and, through the release of immunosuppressive cytokines such as IL‐10 and TGF‐β, fosters a tolerogenic milieu [199]. Moreover, PDGFR (ADAM12) stromal mesenchymal cells supply lipids to TAMs, boosting their metabolic fitness and establishing an efferocytosis–metabolism–immune evasion axis that reinforces the immune barrier [200].

Current approaches target efferocytic receptors to disrupt this negative feedback loop. Pharmacological inhibition of MerTK or AXL, when combined with radiotherapy or chemotherapy, augments the immunogenicity of tumor cell death, increases antigen release, and thereby strengthens adaptive immune activation [201]. Complementarily, PI3Kγ inhibitors dampen TAM‐mediated efferocytosis, permitting residual tumor debris to engage the cGAS–STING pathway, induce type I interferon signaling, and enhance T‐cell recruitment and effector function—effects that potentiate radiotherapy and improve survival in pancreatic cancer [14]. Strategies that purposefully delay clearance to increase antigen exposure must be tuned to avoid necrotic inflammation, thus emphasizing the need to balance immune stimulation with tissue protection.

Nanoparticle delivery systems offer distinct advantages within this framework. As Cabral et al. [202] highlight, nanoplatforms can precisely ferry efferocytosis modulators while co‐releasing tumor‐associated antigens, thereby converting “cold” tumors into “hot, ” increasing immune‐cell infiltration and activation, and ultimately producing more durable antitumor immunity. Efferocytosis‐linked molecules are emerging as the next‐generation immune checkpoint targets. For example, TAMs with high CD276 expression mediate “silent clearance, ” which protects tumor cells, and the antibody blockade of CD276 synergizes with PD‐1 inhibitors [25]. Conversely, the loss of Anxa1 diminishes macrophage efferocytosis yet heightens cGAS–STING activation and immune remodeling [203]. Taken together, these findings indicate that inhibiting efferocytosis does not invariably foster immune escape. In select contexts, calibrated restriction can activate innate immunity and reprogram the tumor immune microenvironment.

In summary, efferocytosis dysfunction is highly context‐dependent and can exert opposing effects in different diseases. In autoimmunity, insufficient efferocytosis sustains antigen exposure and immune activation; in tumors, hyperactive “silent” clearance promotes immune evasion. Therefore, therapeutic strategies should be tailored to the specific pathology and microenvironment: in autoimmunity, bolster protolerogenic efferocytosis to restore tolerance; in cancer, restrain silent efferocytosis; and, when appropriate, delay clearance to enhance immunogenicity. In addition to macrophages, the active role of tumor cells as efferocytosis substrates is often underappreciated. For example, tumor cells secrete high‐mobility group box 1, CRT, and other factors that interfere with PS recognition, enabling escape from immune clearance [199]. Future work should delineate bidirectional “predator–prey” signaling and develop interventions that modulate both sides, providing a mechanistic foundation and translational path for precision therapies in autoimmunity and cancer.

## 5 From Mechanism to Medicine: Clinical Translation of Efferocytosis

Defects in efferocytosis are closely linked to the pathogenesis of various diseases, highlighting the potential of targeting these processes as a promising clinical strategy. This section reviews recent advancements in the use of key efferocytic proteins, such as MerTK, Axl, and GAS6, as clinical biomarkers for cardiovascular diseases, cancer, and inflammatory disorders. We also discuss the emerging therapeutic approaches designed to enhance efferocytosis, including small‐molecule drugs, biologics, and nanoparticle‐based delivery systems, along with early clinical findings. Finally, we examine the current status of clinical translation, focusing on the challenges that remain in implementing these strategies in clinical practice.

### 5.1 Advances in Clinical Biomarkers

**FIGURE 5:**
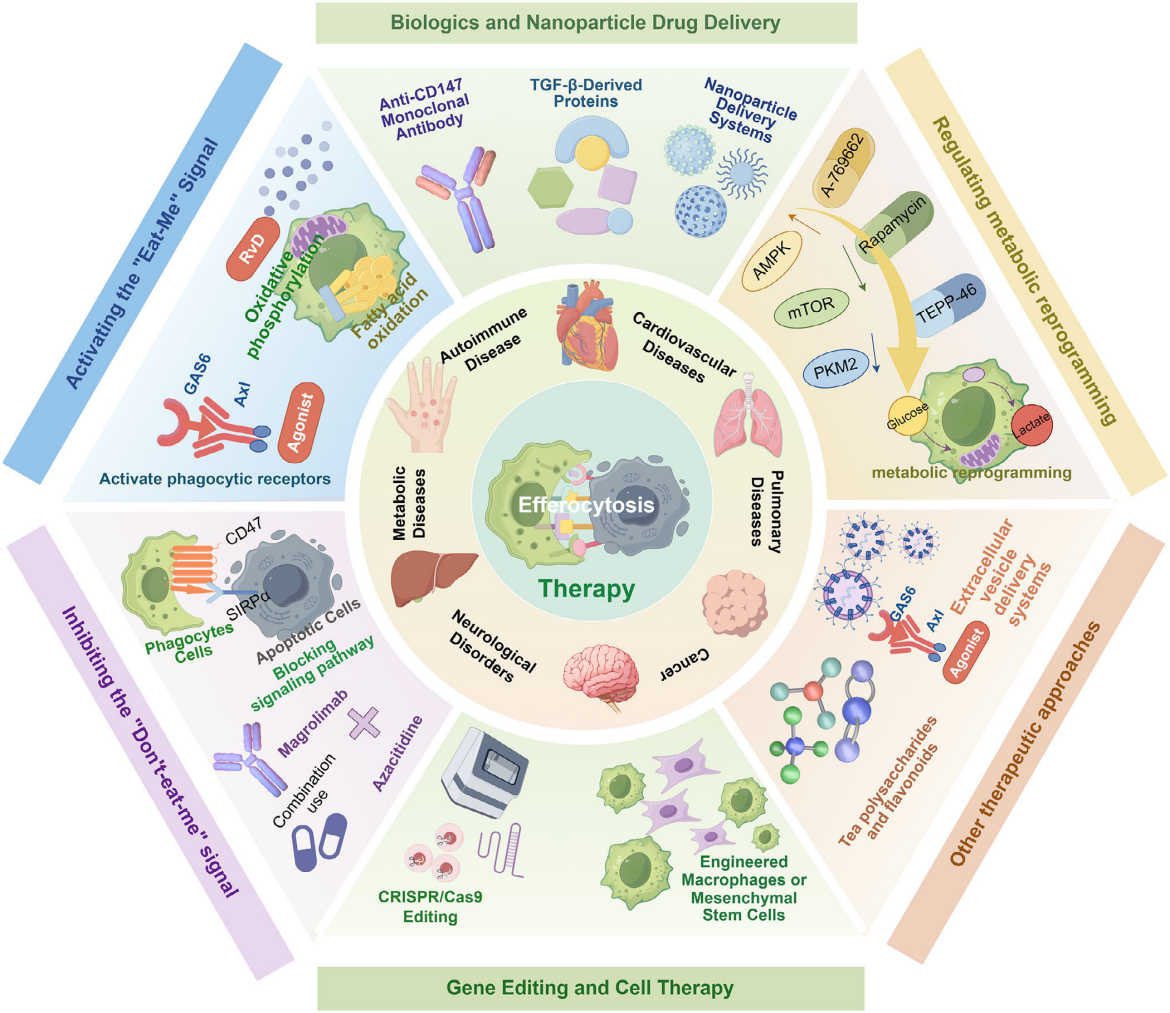
Therapeutic strategies targeting efferocytosis. Modulating efferocytosis can be achieved through diverse approaches. (a) Activating “eat‐me” signals: small‐molecule agonists (e.g., PPARγ ligands, resolvins) or engineered proteins such as GAS6 enhance apoptotic cell recognition and clearance. (b) Inhibiting “don't‐eat‐me” signals: blockade of CD47–SIRPα (e.g., magrolimab) restores efferocytosis and is under clinical evaluation. (c) Gene editing and cell therapy: CRISPR/Cas9 and engineered macrophages or stem cells augment clearance and reprogram tissue immunity. (d) Biologics and nanoparticles: antibodies, TGF‐β‐derived proteins, and SPMs promote efferocytosis, while nanoparticles enable targeted delivery. (e) Regulating metabolism: modulation of AMPK, mTOR, or PKM2 pathways improves metabolic reprogramming and supports serial efferocytosis. (f) Other approaches: combining efferocytosis modulation with immunotherapy or radiotherapy refines immune responses, with selective inhibition enhancing tumor immunogenicity. Collectively, these strategies position efferocytosis as a versatile therapeutic target in cardiovascular, pulmonary, neurological, metabolic, autoimmune diseases, and cancer. *Abbreviations*: PPARγ, peroxisome proliferator‐activated receptor‐γ; GAS6, growth arrest‐specific 6; SIRPα, signal regulatory protein‐α; CRISPR, clustered regularly interspaced short palindromic repeats; MerTK, MER proto‐oncogene tyrosine kinase; AMPK, AMP‐activated protein kinase; mTOR, mammalian target of rapamycin; PKM2, pyruvate kinase M2; SPMs, specialized proresolving mediators; TGF‐β, transforming growth factor‐β.

As our understanding of efferocytosis deepens, therapeutic strategies targeting this process are being explored (Figure 5). Its receptors and ligands are emerging as clinical biomarkers for disease assessment and therapeutic guidance in chronic disorders. This link is most evident in cardiovascular diseases, cancer, and inflammatory conditions, in which an imbalance in the efferocytic program is correlated with disease onset, progression, and prognosis. For instance, MerTK, the best‐studied efferocytic receptor, is downregulated in AS and is associated with the expansion of the necrotic core, indicating prognostic value [22]. In contrast, within the tumor microenvironment, tumor‐derived factors upregulate Axl and MerTK, and such overexpression is linked to immune evasion and treatment resistance, as has been reported for bladder cancer and other malignancies [25]. Beyond receptors, ligand‐level changes are informative; loss of CD147 stimulates macrophage secretion of GAS6, which enhances the clearance of late apoptotic cells and supports GAS6 as a biomarker reflecting immune burden and phagocytic dynamics [204]. Collectively, these observations highlight the efferocytic components as tractable biomarkers across diverse disease settings and motivate pathway‐level monitoring rather than single‐node readouts. They also help to define the therapeutic entry points discussed below.

However, most available biomarkers interrogate only a single step, recognition or engulfment, which makes it difficult to capture pathway dynamics. A recent framework partitions efferocytosis into three stages—recognition, engulfment, and digestion–metabolism—and advocates longitudinal monitoring to more accurately assess inflammatory‐resolution capacity and therapeutic response. At the recognition stage, PS externalization functions as an early “eat‐me” cue, but its transient kinetics narrow the clinical detection window [205]. To extend this window, the GAS6–Axl axis—which remains persistently upregulated after PS exposure, can be assayed, and plasma Axl levels or single‐cell RNA sequencing (scRNA‐seq) can be used to track tumor burden and treatment response [136]. This stage‐resolved approach links specific readouts to distinct biological steps and sets up marker selection for downstream phases.

Within this stage‐resolved framework, engulfment stage biomarkers center on MerTK, the expression of which is strongly correlated with phagocytic efficiency. Under repeated bacterial stimulation, a KLF4‐driven MerTK^hi^ subset of alveolar macrophages expands in proportion to engulfment capacity [204]. To capture these dynamics and their metabolic coupling, multiomics platforms, such as mass cytometry and single‐cell transcriptomics, can simultaneously profile Axl, MerTK, and the metabolic programs they regulate, enabling fine‐grained functional tracking [25]. Finally, at the digestion–metabolism stage, the NADPH/ATP ratio reflects lysosomal degradative efficacy and cellular energy status, providing a quantitative readout of digestion‐phase activity [206].

### 5.2 Drugging Efferocytosis: Receptor Agonists, Biologics, and Nanomedicine

Building on these biomarker‐defined pathway nodes, and given the central role of efferocytosis in controlling inflammation and promoting tissue repair, multitarget strategies acting across recognition, engulfment, and digestion/metabolism have advanced rapidly, spanning small‐molecule agonists, biologics, and nanoparticle delivery platforms. At the receptor level, positive allosteric modulators of MerTK and Axl increase receptor autophosphorylation, boost phagocytic efficiency, and reduce necrotic core size in AS models [27]. In parallel, magrolimab—a CD47–SIRPα‐blocking antibody that disables the “DON'T‐EAT‐me” signal—has advanced to Phase II testing; antibody–small‐molecule conjugate formats built on this axis have triggered secondary efferocytic responses in solid tumors and strengthened antitumor immunity [27].

A complementary approach targets postefferocytic metabolic reprogramming as an actionable therapeutic node. Activating AMPK with A‐769662 enhanced fatty acid oxidation, restored MerTK expression, and reduced vascular inflammation in high‐fat‐diet models [100]. Inhibition of the mTOR axis by rapamycin blocks Rheb–mTORC1 signaling, thereby sustaining efferocytosis and promoting cholesterol efflux [100]. Targeting glycolysis is also effective: TEPP‐46, an allosteric inhibitor of PKM2, prevents its nuclear translocation and upregulates LRP1, strengthening serial efferocytosis and shrinking atherosclerotic lesions by ∼30% in LDLR^−/−^ mice [207].

Biologics are an orthogonal means of augmenting efferocytosis. An anti‐CD147 monoclonal antibody enhances macrophage phagocytosis in ApoE^−/−^ models and has completed a phase I safety evaluation [204]. Tissue‐targeted TGF‐β‐derived proteins accumulate at lesion sites and induce MerTK, yielding more durable PD effects than free TGF‐β [27]. High‐affinity engineered GAS6 promotes phagocytosis and attenuates inflammation in arthritis models [194]. Structurally optimized SPMs activate the MerTK–Arg1 pathway while suppressing glycolysis, thereby reprogramming inflammatory responses [100]. Consistent with these biological approaches, an ongoing Phase II trial indicated that Pep2–8 mitigates arterial aging by restoring endothelial MerTK‐dependent efferocytosis [208].

Finally, delivery technologies can increase the precision and durability of these interventions. Because key efferocytic receptors are enriched in the plasma membrane, they are attractive targets for nanodelivery. For example, mesenchymal stem cells (MSC‐EVs) cell‐enriched GAS6 effectively enhances macrophage efferocytosis both in vivo and in vitro through the GAS6/MerTK/ERK/COX2 signaling pathway [209]. Anxa1 is also key to efferocytosis, and nanoliposomes loaded with Anxa1 mRNA alleviate inflammation by restoring efferocytosis in macrophages [210]. Beyond receptor targeting, metabolic nodes can guide cargo selection. For example, PFKFB2 drives a lactate‐dependent program that sustains serial efferocytosis [178]. Building on these principles, virus‐like nanoparticles delivering BELMO and related effector genes enable the sustained release of prophagocytic cues at lesion sites and, when paired with near‐infrared probes, allow for real‐time imaging of the phagocytic process [211]. Innovative noninvasive systems have further expanded these approaches. An ultrasound‐driven electric conversion hydrogel (SHG@GBT) modulates macrophage efferocytosis, thereby optimizing the immune microenvironment and promoting tissue regeneration [212]. This system integrates piezoelectric heterojunctions with a reorganized hydrogel matrix to convert ultrasound energy into localized electrical signals. These signals activate macrophage calcium channels, increase calcium influx, and enhance efferocytosis, angiogenesis, and collagen synthesis. Collectively, these technological advances have established the foundation for clinical translation.

### 5.3 Clinical Readiness of Efferocytosis Modulation: Evidence, Gaps, and Solutions

Translating these advances and as basic research advances, strategies to modulate efferocytosis are moving into clinical testing. Early human studies suggested the potential to recalibrate immune homeostasis and promote tissue repair [213]. For example, after intravenous infusion, mesenchymal stromal cells undergo rapid apoptosis in the lungs and are subsequently cleared by alveolar macrophages, which reprogram the local metabolic and inflammatory pathways, yielding favorable immunoregulatory effects [214]. Collectively, these observations support the feasibility of dual‐target interventions that induce apoptosis and enhance clearance.

Despite these encouraging findings, the complexity of efferocytosis poses substantial hurdles for its clinical translation. First, the absence of standardized histological and molecular endpoints hampers cross‐center comparability and rigorous benchmarking of trial outcomes [215]. Second, because efferocytosis arises from dynamic interactions between apoptotic cells and phagocytes, conventional PK/PD frameworks poorly capture its spatiotemporal kinetics—a limitation analogous to the coupled “replication–clearance” dynamics that complicate phage therapy [216]. Third, commonly used acute animal models fail to reproduce the trajectory of human chronic diseases and pronounced interspecies differences further erode confidence in efficacy extrapolation [217]. Together, these factors slow the translation of efferocytosis‐centered interventions from the bench to bedside.

To address these barriers, advances in multiomics (e.g., single‐cell sequencing, spatial omics, and metabolomics) and cross‐disciplinary methods will enable systematic evaluation and dynamic modeling of efferocytosis [218] and clearance of dead cells by efferocytosis. Establishing standardized assessment frameworks and metrics and pairing them with refined disease models and nanotracing tools should collectively accelerate the translation of efferocytosis‐modulating strategies from discovery to precise, controllable, and reproducible clinical applications.

## 6 Technologies and Models Advancing Efferocytosis Research

In cardiovascular, neurological, pulmonary, and metabolic diseases, defects in efferocytosis drive persistent inflammation, impair tissue repair, and disrupt immune homeostasis, underscoring its increasing pathological importance. However, because efferocytosis is highly dynamic and is regulated by multiple inputs, its context‐specific mechanisms, spatiotemporal features, and regulatory nodes remain incompletely defined. Conventional approaches are limited in three areas: resolving phagocyte heterogeneity, capturing real‐time “eat‐me/don't‐eat‐me” signaling dynamics, and delineating crosstalk between efferocytosis, metabolism, and immune networks [219].

**FIGURE 6:**
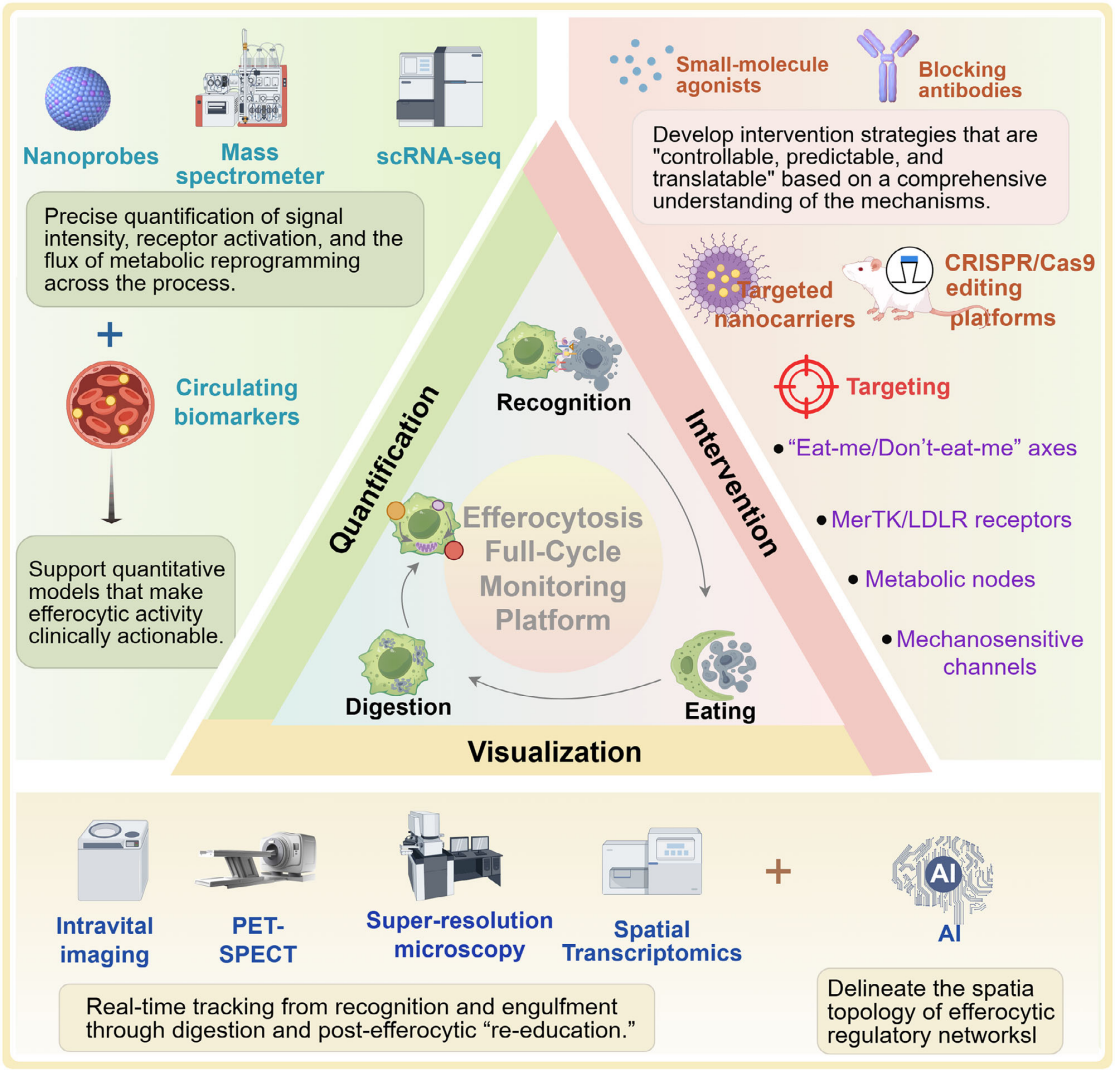
A full‐cycle monitoring platform for efferocytosis. Efferocytosis can be systematically studied through three interconnected modules. (a) Quantification: nanoprobes, mass spectrometry, single‐cell multiomics, and circulating biomarkers enable precise measurement of receptor activation, signaling flux, and metabolic reprogramming, supporting clinically actionable models of efferocytic activity. (b) Visualization: advanced imaging, including super‐resolution and intravital microscopy, PET/SPECT, and spatial transcriptomics, provides dynamic tracking of apoptotic cell clearance. AI‐assisted analysis delineates spatial networks of efferocytic regulation. (c) Intervention: mechanism‐based strategies target “eat‐me/don't‐eat‐me” checkpoints, MerTK/LDLR receptors, and metabolic nodes. Tools include small‐molecule agonists, blocking antibodies, targeted nanocarriers, and CRISPR/Cas9‐based editing. This full‐cycle platform enables dynamic monitoring of efferocytosis, facilitates biomarker discovery, and provides a basis for precision therapeutic intervention. *Abbreviations*: PET, positron emission tomography; SPECT, single‐photon emission computed tomography; AI, artificial intelligence; LDLR, low‐density lipoprotein receptor; MerTK, MER proto‐oncogene tyrosine kinase; CRISPR, clustered regularly interspaced short palindromic repeats.

To address these gaps, rapid advances in high‐resolution microscopy, single‐cell and spatial omics, organoid modeling, CRISPR‐based editing, and AI have expanded the investigative toolkit [27, 220]. Multiscale multimodal integration is moving the field toward system‐level precision while providing platforms for individualized diagnostics and targeted interventions. The following sections review the key frontiers—live imaging and tracking, omics‐based dissection, functional disease models, and AI‐enabled analytics—and outline the directions for future development (Figure 6).

### 6.1 Super‐Resolution and Intravital Imaging: Reconstructing the Spatiotemporal Dynamics of Phagocytosis

Super‐resolution microscopy (SRM) surpasses the diffraction limit, enabling nanometer‐scale analysis of phagocyte ultrastructure and dynamics during efferocytosis. Among widely used modalities, stimulated emission depletion (oSTED), structured illumination (SIM), and single‐molecule localization (PALM/STORM) offers complementary strengths [221]. oSTED employs a ring‐shaped depletion beam to shrink the point‐spread function and, with time gating, markedly lowers the background [221], enabling in vivo tracking of macrophage F‐actin leading‐edge rearrangements and providing real‐time evidence that the MAS/KLF4/MERTK axis regulates efferocytic efficiency [141]. With a high scan speed and low phototoxicity, SIM supports the longitudinal imaging of lipid droplet trafficking after phagocytosis and quantification of lysosomal upregulation kinetics [222]. PALM/STORM localizes individual fluorophores with ∼20 nm precision, resolves adhesion–cytoskeletal architecture, and reveals podosome remodeling after phagocytic activation [223]. Collectively, these approaches reconstructed the spatiotemporal choreography of efferocytosis from cytoskeletal remodeling to cargo processing and established a subcellular foundation for multiscale analyses.

Complementing these subcellular views, intravital imaging with multiphoton and light‐sheet microscopy links single‐cell behavior to whole‐organ physiology. In catch‐up transgenic mice, lung intravital imaging shows that neutrophils are cleared locally by efferocytosis after viral infection, which modulates downstream T‐cell responses and highlights the central role of local efferocytosis in immune homeostasis [224]. Similarly, in AS models, in vivo microsphere tracking combined with confocal intravital microscopy revealed that plaque macrophages remain spatially stable while cellular corpses accumulate, a pattern that correlates with necrotic core expansion [225]. Looking ahead, integrating next‐generation light‐sheet imaging with deep‐learning‐assisted 3D reconstruction and coupling these organ‐scale readouts with SPM‐derived subcellular metrics is expected to extend intravital imaging to large animals and clinical endoscopic platforms, enabling precise evaluation of patient‐specific immunointerventions.

### 6.2 Multiomics Integration: Dissecting Efferocytic Heterogeneity and Metabolic Regulatory Networks

The rapid expansion of multiomics technologies has substantially deepened our understanding of the spatial organization of efferocytosis, the diversity of phagocyte lineages, and the mechanisms of metabolic reprogramming. scRNA‐seq is the primary tool for resolving the heterogeneity among phagocyte subpopulations [226]. In pancreatic ductal adenocarcinoma, Zhu et al. [227] identified a transglutaminase 2^+^ (TGM2^+^) macrophage subset with heightened efferocytic activity. This state co‐occurs with the activation of hypoxia and glycolytic programs, indicating a tight coupling between efferocytosis and cellular metabolism. Similarly, in MI models, Trem2^hi^ macrophages enriched in ischemic regions display strong efferocytic signatures [228]. Together, these single‐cell analyses illustrate how disease context shapes efferocytic phenotypes and their metabolic wiring.

Complementing single‐cell analyses and spatial transcriptomics by preserving tissue architecture and enabling localized transcript profiling maps the intratumoral enrichment of efferocytosis‐related receptors, such as MERTK and AXL [229]. In diabetic wound models, high SLC7A11 expression is detected in dendritic cells, which act as a molecular brake in efferocytosis [230].

At the metabolite level, metabolomic and lipidomic profiling, especially using high‐throughput LC–MS/MS and GC, can identify metabolites that index efferocytic activity. For instance, persistent elevation of PGE2 in efferocytosis‐associated reparative microenvironments supports its use as an endogenous tracer of tissue regeneration [231]. Integrating scRNA‐seq, spatial omics, and metabolomics will clarify how transcriptional programs couple with metabolism during efferocytosis.

### 6.3 | Animal and Organoid Models: Expanding Functional Validation in Disease Contexts

To investigate efferocytosis across disease settings, genetically engineered models are foundational for dissecting the mechanisms of efferocytic impairment and testing targeted interventions. In cancer and AS models, perturbing MerTKvia neutralizing antibodies, small‐molecule inhibitor UNC2025, or gene knockout consistently enhances antitumor immunity or reduces necrotic‐core formation [232, 233, 234]. Knockouts of key regulators, including GAS6, Arg1, ODC, and WDFY3, have also demonstrated their essential contributions to efferocytic dysfunction [113, 235, 236]. Beyond direct receptor manipulation, modulating upstream controllers such as CD147 can indirectly augment efferocytosis, thereby broadening the therapeutic target space [204]. Complementing these in vivo systems, organoid platforms (detailed below) provide human‐relevant, controllable settings to investigate efferocytic dynamics under defined cues and bridge species‐specific gaps in mechanisms and pharmacology.

Specifically, organoid systems preserve the three‐dimensional architecture and cellular heterogeneity, providing physiologically relevant in vitro platforms for probing efferocytosis dynamics. In intestinal organoids, donor age stratifies efferocytic capacity, implicating age‐dependent control of “eat‐me” signaling [237]. Glioma‐like brain organoids recapitulate hypoxia‐necrotic microenvironments, and the pharmacological inhibition of TGM2 markedly reduces macrophage efferocytosis, underscoring the utility of brain organoids for drug screening [238]. Collectively, these examples demonstrate how organoids couple mechanistic dissections with preclinical testing in human‐relevant contexts.

Simultaneously, CRISPR/Cas9 is increasingly used to modulate efferocytosis. Targeted editing of key nodes—including MerTK, CD47, and SIRPα—enhances phagocytic capacity and potentiates antitumor immunity in preclinical tumor models [239, 240, 241]. Complementing these efforts, stimulus‐responsive editing systems, such as lactate‐triggered nanoplatforms, provide spatiotemporal control of efferocytosis pathways and open new therapeutic avenues. Together, these strategies couple precise gene manipulation with context‐dependent modulation, thereby strengthening the translational pipelines.

### 6.4 AI‐Enabled Approaches: Data‐Driven Mechanism Discovery and Target Identification

Deep learning underpins automated recognition and structural/semantic segmentation of efferocytosis imaging datasets [242]. CNN architectures, most notably U‐Net, enable the robust detection and quantitative tracking of phagocytic cups and phagosomes, markedly increasing the accuracy and throughput of image analyses [243]. Beyond imaging, integrative AI multiomics pipelines help to decode the regulatory networks that govern efferocytosis [244]. Combining scRNA‐seq with non‐negative matrix factorization and CNN‐based classifiers revealed macrophage subsets that reprogram the tumor immune microenvironment in primary and metastatic melanoma, informing patient stratification and prognosis [245]. In drug discovery, AI‐assisted virtual screening prioritizes small molecule ligands and maps signaling routes for efferocytic receptors, accelerating rational design [246]. As datasets expand and algorithms mature, AI approaches are poised to advance efferocytosis research end‐to‐end, from biomarker discovery and target identification to screening, thereby shortening the path from basic insights to clinical applications (Table 1).

**TABLE 1:** Efferocytosis‐related drug development: potential therapeutic targets.

Drugs	Categorization	Description	Target	Disease	Phase	References
SPI@hEL–RS17	Recognition	RS17 peptide blocked the CD47–SIRPα signaling in 4T1‐tumor‐bearing mice.	CD47–SIRPα	Breast cancer	—	[43]
RLTR–Wnt2@ExoCD47	Recognition	In mouse models of liver injury induced by APAP and DMN, RLTR–Wnt2@ExoCD47 delivers the drug Wnt2 by blocking CD47 to prevent phagocytic cells from engulfing it.	CD47	DILI	—	[45]
Magrolimab	Recognition	Magrolimab enhances the phagocytosis of cancer cells in patients with AML who are ineligible for intensive induction chemotherapy, including those with TP53 mutations.	CD47	AML	II	[62]
B‐LNP/diABZI	Recognition	In the brains of glioma‐bearing mice, B‐LNP blocks CD47 and enhances the phagocytic activity of TAMs, promoting tumor regression.	CD47	Glioblastoma	—	[66]
R@MLP	Recognition	R@MLP enhances efferocytosis in male ApoE^−/−^ mice and shifts macrophages to an anti‐inflammatory state.	CD47–SIRPα	Atherosclerosis	—	[150]
Anti‐CD147 monoclonal antibody	Recognition	Anti‐CD147 monoclonal antibody promotes efferocytosis and reduces inflammation in ApoE^−/−^ mice.	CD147	Atherosclerosis	I	[204]
MSC–EV–GAS6	Recognition	In the HIRI mouse model, MSC‐EVs enriched with GAS6 effectively enhance macrophage efferocytosis through the GAS6/MerTK/ERK/COX2 signaling pathway and significantly reduce liver injury.	MerTK	HIRI	—	[209]
AL002c	Eating	AL002c promotes Aβ clearance in mice by upregulating the expression of the TREM2 phagocytic receptor.	TREM2	Alzheimer's disease	I	[163]
Resolvin D1	Eating	RvD1 enhances microglial phagocytosis of neutrophils, reducing neutrophil accumulation and alleviating neuroinflammation in the I/R brain injury mouse model.	Metabolic reprogramming	Ischemic stroke	—	[173]
Eldecalcitol	Eating	Eldecalcitol promotes efferocytosis in a rat model of DPD, facilitating inflammation resolution and tissue repair.	—	DPD	—	[187]
TEPP‐46	Eating	TEPP‐46 reduces atherosclerosis in LDL^−/−^ mice by promoting macrophage efferocytosis via downregulating PKM2 expression.	PKM2	Atherosclerosis	—	[207]
Pep2–8	Eating	Pep2–8 enhances efferocytosis in aged mice by upregulating MerTK, thereby alleviating vascular aging.	MerTK	—	II	[208]
ACM@U‐FGF21	Digestion	In mice with LPS‐induced ALI, ACM@U‐FGF21 inhibits macrophage phagocytosis and subsequent polarization to an inflammatory state.	—	ALI	—	[178]

*Abbreviations*: AML, acute myeloid leukemia; DILI, drug‐induced liver injury; LPS, lipopolysaccharide; ALI, acute lung injury; DPD, diabetic periodontitis; HIRI, hepatic ischemia–reperfusion injury; APAP, acetaminophen; DMN, dimethylnitrosamine.

## 7 Conclusion and Prospects

By silently clearing apoptotic cells, efferocytosis preserves tissue homeostasis and sustains immune tolerance. Therefore, it is central to host defense, immune regulation, and tissue repair. This review synthesizes the molecular logic of each stage—recognition, engulfment, and digestion—together with the accompanying metabolic reprogramming, and delineates the roles of efferocytosis in cardiovascular, neurological, pulmonary, metabolic, and oncological diseases. We further emphasize the coupling between postefferocytic metabolic rewiring and immune control. On this basis, we propose the full‐cycle, dynamic monitoring of efferocytosis as a foundation for precision interventions across organ systems. Concurrent advances in high spatiotemporal resolution imaging, multiomics profiling, and AI‐enabled analytics are expanding experimental capabilities and accelerating translation from mechanism to clinic.

Despite major advances, several fundamental questions persist regarding mechanisms, disease contexts, and methodologies. Mechanistically, the molecular determinants of efferocytic selectivity remain unclear and the mechanism by which phagocytes prioritize concurrent apoptotic targets is largely unknown. The circuitry coordinating “eat‐me” and “don't‐eat‐me” cues is also only partially mapped, and its temporal dynamics—especially in tumor immune evasion and autoimmunity—require deeper study. In addition, high‐resolution real‐time tools to trace how postefferocytic signaling programs phagocyte fate and toggle immune tolerance versus activation are lacking. At the disease level, efferocytic function is organ‐specific and time‐dependent; however, routine clinical practice still lacks high‐sensitivity biomarkers or imaging modalities for dynamic assessment. Methodologically, current imaging and omics platforms are constrained by temporal and spatial resolution, throughput for multimodal integration, and in vivo operability. Collectively, these gaps limit mechanistic inferences and slow clinical translations.

Breakthroughs in efferocytosis research should focus on three mutually reinforcing objectives: quantification, visualization, and intervention. Integrating multimodal tools into a full‐cycle dynamic monitoring platform—nanoprobes targeting PS or MerTK or reporting metabolic flux ratios, mass cytometry, and single‐cell multiomics—would enable the precise quantification of signal intensity, receptor activation, and flux of metabolic reprogramming across the process. Coupling circulating biomarkers (e.g., GAS6 and SPMs) with clinical trajectories and treatment responses can support quantitative models that make efferocytic activity clinically actionable.

Complementary measurements, advanced imaging, and spatial profiling can render the process end‐to‐end visible. Super‐resolution and multiphoton intravital imaging, PET/SPECT molecular imaging, and spatial omics allow real‐time tracking from recognition and engulfment through digestion and postefferocytic “re‐education.” These approaches can map the spatial distribution, migratory routes, and interactions of distinct phagocyte subsets and apoptotic targets. When integrated with AI‐assisted 3D reconstruction and dynamic modeling, they delineate the spatial topology of efferocytic regulatory networks and deepen mechanistic resolution.

Building on these readouts, intervention became the third pillar. Mechanistically informed strategies can target the “eat‐me/don't‐eat‐me” axes; MerTK/LDLR receptors; metabolic nodes (e.g., PFKFB2, itaconate); and mechanosensitive channels (e.g., Piezo1, TRPM7). Practical implementation spans small‐molecule agonists, blocking antibodies, targeted nanocarriers, and CRISPR/Cas9 editing platforms. Engineered macrophage or MSC therapies and proefferocytic agents should be advanced from animal models to early‐phase clinical studies to realize a tangible translational impact. Collectively, these three pillars—measurement, visualization, and intervention—provide a coherent pathway from the mechanism to the clinic.

In summary, efferocytosis is a pivotal nexus between cell death and immune homeostasis and has substantial translational potential in chronic inflammatory and immune disorders as well as cancer. To realize this potential, future studies should elucidate and manipulate spatiotemporal regulation by integrating multidisciplinary technologies to progress from mechanistic insight to precise modulation, thereby accelerating the development of clinically useful diagnostics and therapies.

## Author Contributions

Chaofu Li and Yukun Yang: Conceptualization (lead); writing – original draft (lead); writing – review and editing (lead). Fating Zhou and Qiuyan Jiang: Conceptualization (supporting); writing – original draft (supporting); writing – review and editing (supporting). Yingying Jiang, Xuanjie Huang, Yiqiong Zhang, Zhengmeng Ye, Gang Xu, Guoying Kao, and Ke Zhou: Project administration (supporting); writing – review and editing (supporting). Fan Yang and Jun Xiao: Conceptualization (supporting); project administration (supporting); resources (lead). Wei Wu and Chuanwei Li: Conceptualization (supporting); project administration (lead); resources (lead); writing – original draft (supporting); writing – review and editing (equal). All authors have read and approved the final version of the manuscript.

## Funding

We gratefully acknowledge the support provided by the following research grants: Chongqing Key Laboratory of Emergency Medicine (Grant No. 2024KFKTYB01), a key project co‐organized by the Health Commission and the Science & Technology Bureau of Chongqing Province (Grant No. 2024DXM024), Natural Science Foundation of Chongqing Municipality (Grant No. CSTB2023NSCQMSX0348), the National Natural Science Foundation of China (Grant No. 82500355), and the Science and Technology Research Program of Chongqing Municipal Education Commission (Grant No. KJQN202300114).

## Ethics Statement

The authors have nothing to report.

## Conflicts of Interest

The authors declare no conflicts of interest.

## Data Availability

The authors have nothing to report.
